# KIAA1429-mediated m6A modification of CHST11 promotes progression of diffuse large B-cell lymphoma by regulating Hippo–YAP pathway

**DOI:** 10.1186/s11658-023-00445-w

**Published:** 2023-04-19

**Authors:** Xiaomin Chen, Tiange Lu, Yiqing Cai, Yang Han, Mengfei Ding, Yurou Chu, Xiangxiang Zhou, Xin Wang

**Affiliations:** 1 Department of Hematology, Shandong Provincial Hospital, Shandong University, No.324, Jingwu Road, Jinan, 250021 Shandong China; 2 Department of Hematology, Shandong Provincial Hospital Affiliated to Shandong First Medical University, Jinan, 250021 Shandong China; 3 Shandong Provincial Engineering Research Center of Lymphoma, Jinan, 250021 Shandong China; 4 Branch of National Clinical Research Center for Hematologic Diseases, Jinan, 250021 Shandong China; 5 National Clinical Research Center for Hematologic Diseases, The First Affiliated Hospital of Soochow University, Suzhou, 251006 China

**Keywords:** Diffuse large B-cell lymphoma, *N*^6^-methyladenosine, KIAA1429, YTHDF2, CHST11, Hippo–YAP

## Abstract

**Background:**

*N*^6^-methyladenosine (m6A) has been shown to participate in various essential biological processes by regulating the level of target genes. However, the function of m6A modification mediated by KIAA1429 [alias virus-like m6A methyltransferase-associated protein (VIRMA)] during the progression of diffuse large B-cell lymphoma (DLBCL) remains undefined.

**Methods:**

The expression and clinical significance of KIAA1429 were verified by our clinical data. CRISPR/Cas9 mediated KIAA1429 deletion, and CRISPR/dCas9-VP64 for activating endogenous KIAA1429 was used to evaluate its biological function. RNA sequencing (RNA-seq), methylated RNA immunoprecipitation sequencing (MeRIP-seq), RNA immunoprecipitation (RIP) assays, luciferase activity assay, RNA stability experiments, and co-immunoprecipitation were performed to investigate the regulatory mechanism of KIAA1429 in DLBCL. Tumor xenograft models were established for in vivo experiments.

**Results:**

Dysregulated expression of m6A regulators was observed, and a novel predictive model based on m6A score was established in DLBCL. Additionally, elevated KIAA1429 expression was associated with poor prognosis of patients with DLBCL. Knockout of KIAA1429 repressed DLBCL cell proliferation, facilitated cell cycle arrest in the G2/M phase, induced apoptosis in vitro, and inhibited tumor growth in vivo. Furthermore, carbohydrate sulfotransferase 11 (CHST11) was identified as a downstream target of KIAA1429, which mediated m6A modification of *CHST11* mRNA and then recruited YTHDF2 for reducing *CHST11* stability and expression. Inhibition of CHST11 diminished MOB1B expression, resulting in inactivation of Hippo–YAP signaling, reprogramming the expression of Hippo target genes.

**Conclusions:**

Our results revealed a new mechanism by which the Hippo–YAP pathway in DLBCL is inactivated by KIAA1429/YTHDF2-coupled epitranscriptional repression of CHST11, highlighting the potential of KIAA1429 as a novel predictive biomarker and therapeutic target for DLBCL progression.

**Supplementary Information:**

The online version contains supplementary material available at 10.1186/s11658-023-00445-w.

## Background

Diffuse large B-cell lymphoma (DLBCL) is an aggressive B-cell non-Hodgkin lymphoma with a high degree of heterogeneity [1, 2]. Although approximately 50–70% of patients with DLBCL respond to standard rituximab chemotherapy, the remainder exhibits a refractory or relapsed process [3, 4]. The application of epigenetic drugs, including HDAC inhibitors [5] and EZH2 inhibitors [6], in patients with DLBCL has exerted a crucial role in improving the treatment of this patient population [7], but the efficacy remains limited. Further exploration of the biological functions of epigenetic alterations in DLBCL and their potential as therapeutic targets may contribute to the optimal individualized treatment of patients with DLBCL.

Among eukaryotic messenger RNAs (mRNAs), *N*^6^-methyladenosine (m6A) is the most prevalent epigenetic modification, and its regulation of RNA depends on the dynamic interaction between its methyltransferases (“writers”), demethylases (“erasers”), and binding proteins (“readers”) [8–10]. As an m6A methyltransferase, KIAA1429 [alias virus-like m6A methyltransferase associated (VIRMA)] is considered to be a scaffold for the methyltransferase complex, functioning as a linker between the catalytic core component and the RNA substrate to guide the installation of m6A at specific locations [11, 12]. Growing evidence has indicated that m6A modification is involved in the development of various cancers, including pancreatic cancer [13], liver cancer [14, 15], bladder cancer [16], and gastric cancer [17]. Moreover, KIAA1429 has been reported to serve as a regulator of the TGFβ [18] and TNF pathways [19] to promote tumor progression. Although recent studies have revealed that m6A methyltransferases exhibit oncogenic activity in DLBCL [20, 21], their epigenetic mechanisms remain elusive, especially the biological significance of KIAA1429 and its molecular mechanism in DLBCL have not been elucidated.

Our current study aimed to dissect a novel mechanism of epitranscriptomic regulation in DLBCL. We found that KIAA1429 exhibited high levels of expression in DLBCL and increased m6A level of *CHST11*, thereby recruiting YTHDF2 to inhibit *CHST11* stability and expression, resulting in promotion of DLBCL tumorigenesis. Collectively, this study highlighted the probability of KIAA1429 as a therapeutic target for DLBCL and might provide new insights into formulating individualized treatment strategies.

## Methods

### Clinical specimens and cell lines

Sixty patients with de novo DLBCL and 20 patients with reactive hyperplasias of lymph nodes (RHL) as controls were recruited with informed consent, and lymph node specimens were collected. Blood samples were collected from healthy donors to obtain peripheral blood mononuclear cells (PBMCs). CD19^+^ magnetic microbead kit (Miltenyi Biotec, Bergisch Gladbach, Germany) was used to isolate and purify normal B cells from isolated PBMCs. The Medical Ethics Committee of Shandong Provincial Hospital approved all studies, and each participant provided informed consent based on the Helsinki Declaration. We cultured human DLBCL cell lines OCI-LY1, OCI-LY8, OCI-LY3, VAL, and U2932 in Iscove’s Modified Dulbecco’s Medium (Gibco, CA, USA) containing 10% fetal bovine serum (FBS) (HyClone, UT, USA) and 1% penicillin–streptomycin at 37 °C and 5% CO_2_. ITD, the inhibitor of TGF-β to block phosphorylation of Smad2 and Smad3 by TGFβ2, was purchased from Selleck (Shanghai, China, S6713).

### Construction of cell lines with stable knockdown, knockout, and overexpression

Lentivirus vectors encoding a control short hairpin RNA (shRNA) or shRNAs against *KIAA1429*, *CHST11*, *YTHDF2*, *YAP*, and *YTHDF2* lentiviral overexpression vectors were purchased from GeneChem (Shanghai, China). KIAA1429 knockout (KO) in DLBCL cells was established by the CRISPR/Cas9 genomic editing system based on lentivirus from SyngenTech (Beijing, China). The CRISPR/dCas9-VP64 gene activation system (SyngenTech) was used to activate endogenous KIAA1429 in DLBCL cells, followed by transfecting with lentivirus and selecting by 5 μg/ml puromycin (AMRESCO, HY-B1743S) after 72 h, where KIAA1429 knockout cells were further isolated for monoclonal cells by limiting dilution and then continued to be amplified. The sequences are presented in Additional file 1: Table S1.

### Cell proliferation assay

Cell Counting Kit-8 (CCK-8) assay (Dojindo, Japan) was used to assess cell proliferation. Ninety-six-well plates were seeded with 1 × 10^4^ DLBCL cells per well for 24–72 h and then incubated with 10 μl of CCK-8 per well for 3 h. Measuring absorbance at 450 nm by the Multiskan GO Microplate Reader (Thermo Scientific, USA).

### Flow cytometric analysis

Cell cycle and apoptosis were determined by flow cytometric analysis on a Navios Flow Cytometer (Beckman Coulter, CA, USA). For apoptosis analysis, we harvested and washed DLBCL cells twice with cold PBS and resuspended cells in 1× binding buffer, followed by adding 5 μl of Annexin V-PE and 5 μL of 7-AAD (BD Biosciences, MA, USA, 559763). Flow cytometry was used to analyze cells after incubation in the dark for 15 min. For cell cycle analysis, a 70% ethanol fixation at −20 °C overnight was followed by washing the cells with PBS and staining them with PI/RNAse stain (BD Biosciences, 550825) for 15 min before flow cytometry analysis was performed.

### m6A methylation assay

DLBCL cells were extracted of 200 ng total RNA, and the m6A RNA methylation level was determined by an m6A RNA methylation quantification kit (Epigentek, NY, USA, P-9005-48). A standard curve was prepared at concentrations from 0.01 to 0.5 ng/µl. Incubation was conducted at 37 °C for 90 min after the RNA samples were evenly spread on the bottom of the indicated plate. For each well, 60 min incubation at room temperature with a capture antibody and 30 min with a detection antibody. Then, each well was supplemented with enhancer solution and incubated for 30 min, followed by adding the assay solution (100 μl) and incubating in the dark for 10 min. The enzymatic reaction was quenched with 100 μL of stop solution, and then absorbance was obtained at 450 nm within 15 min using a microplate reader. Calculating the amount of m6A as m6A (ng) = (sample OD − NC OD)/slope.

### Dual-luciferase reporter assay

OCI-LY1 and U2932 cells with control, KIAA1429 knockdown, and KIAA1429 overexpression were transfected with luciferase reporter pmirGLO-*CHST11*-WT or pmirGLO-*CHST11*-MUT, respectively. After 48 h of transfection, the cells were harvested and analyzed with a Dual-Luciferase Reporter Assay System (Promega, Madison, USA) according to the manufacturer’s instructions. The activity ratio between firefly luciferase and Renilla luciferase was calculated to determine the relative luciferase activity.

### RNA immunoprecipitation assay

RNA immunoprecipitation (RIP) was conducted using an RNA immunoprecipitation kit (BersinBio, bes5101, China) following the manufacturer’s instructions. Briefly, DNA was removed after cell lysis to obtain 1.7 ml of lysate, 0.1 ml of which was used as input, and the remaining lysate was separated into two equal groups. KIAA1429-specific antibodies (Proteintech, 25712-1-AP) or IgG were incubated with cell lysates coated with magnetic beads at 4 °C for 4 h to overnight. With proteinase K digestion buffer, RNA–protein complexes were washed. Purification of immunoprecipitated RNA was undertaken using phenol:chloroform:isoamyl alcohol (125:24:25), followed by assessing the concentration and quality of RNA using a NanoDrop ND-1000. KIAA1429-bound RNA was detected by qPCR, and the corresponding gene enrichment was calculated by normalizing it to the input.

### RNA pulldown

Streptavidin magnetic bead was applied to bind the biotin-labeled *CHST11* probe (GenePharma, Shanghai) after washing. Cell lysates were collected with standard IP lysis buffer (Bersinbio, Bes5102, China). After binding RNA, cell lysates were incubated with the beads at 4 °C in protein–RNA binding buffer to bind RNA with RNA-binding proteins (RBP). RBP complexes were then collected from the beads through stringent washing and elution, and gathered for examination with western blotting.

### RNA stability assay

To inhibit mRNA transcription, OCI-LY1 and U2932 cells with control, KIAA1429 knockdown, and KIAA1429 overexpression were exposed to 5 μg/ml of the transcription inhibitor actinomycin D (Sigma, A9415). Cells were harvested after 0, 50, 100, and 150 min of incubation, and then total RNA was isolated for qPCR.

### RNA sequencing

Extraction of total RNA from three stable KIAA1429 knockdown OCI-LY1 cells and their relevant nontarget controls was performed using RNAiso Plus (TaKaRa). Library construction and RNA sequencing (RNA-seq) were performed at Novogene (Beijing, China), followed by computational analysis. Differential expression analysis was performed by the DESeq2 R package (1.20.0).

### Methylated RNA immunoprecipitation sequencing

The m6A was sequenced by methylated RNA immunoprecipitation sequencing (MeRIP-seq) at Novogene. Briefly, RNA was extracted from DLBCL cells with control or KIAA1429 knockdown in a total of 300 μg. Using an Agilent 2100 bioanalyzer (Agilent) and simpliNano spectrophotometer (GE Healthcare), RNA concentration and integrity were determined. The immunoprecipitation experiment was carried out with fragments of mRNA (100 nt) incubated with anti-m6A polyclonal antibody (Synaptic Systems) for 2 h at 4 °C. Afterward, NEBNext Ultra RNA Library Prepare Kit for Illumina (New England Biolabs) was used to construct libraries from immunoprecipitated mRNAs or input. Following standard protocols, library preparations were sequenced on an Illumina NovaSeq or HiSeq platform with 150-bp read lengths. Sequencing was conducted with three biological replicates. Annotated genomes and gene models were downloaded directly from the genome website. An index of the reference genome was built using BWA v0.7.12, and clean reads were aligned using BWA mem v0.7.12. A HOMER (version 4.9.1) was used to identify the m6A-enriched motifs of each group. Differential peak calling was performed using the exomePeak R package (version 2.16.0) with parameters of *p* value greater than 0.05 and fold change greater than 1.

### Immunofluorescence assay

DLBCL cells were transferred to slides by cytospin centrifugation, fixed in 4% paraformaldehyde, and permeabilized in 0.5% Triton X-100. Subsequently, slides were blocked with 5% normal goat serum for 1 h and incubated with primary antibodies at 4 °C overnight. The next day, the cells were washed and incubated with the secondary antibody (Invitrogen) for 1 h before mounting with DAPI. The Leica TCS SP8 MP confocal microscope system (Germany) was used to analyze images. The primary antibodies included CHST11 (Invitrogen, PA5-103698), MOB1B (ORIGENE, TA501388S), and YAP (Proteintech,13584-1-AP).

### Coimmunoprecipitation assay

DLBCL cells were harvested and lysed on ice using RIPA buffer (Beyotime, P0013) supplemented with protease inhibitors, followed by quantification with the BCA Protein Detection Kit (Shenergy Biocolor). Overnight incubation with 2 µg of antibody was carried out for cell lysates, with normal IgG as a negative control. After adding Protein A/G Plus-agarose beads, the mixture was incubated for 4 h at 4 °C with gentle rotation. The eluted samples were mixed with SDS-PAGE loading buffer and incubated for 10 min at 100 °C. Immunoprecipitates were analyzed by western blotting using the indicated antibodies.

### Quantitative real-time PCR

TRIzol reagent (TaKaRa, Shiga, Japan) was used to extract total RNA, which was reverse-transcribed to cDNA by an Evo M-MLV RT Kit (Accurate Biology, AG11706), followed by quantitative real-time PCR (RT-qPCR) with an SYBR Green Premix Pro Taq HS qPCR Kit (Accurate Biology, AG11701) on a LightCycler 480II system (Roche). The primers are shown in Additional file 1: Table S2.

### Hematoxylin and eosin and immunohistochemistry staining

Hematoxylin and eosin (H&E) were used to stain sections of 4 μm thickness of mouse tumors fixed in 4% paraformaldehyde and embedded in paraffin for histological examination. For immunohistochemical (IHC) staining, 4-μm-thick tissue sections were cut from formalin-fixed paraffin-embedded tissue blocks, deparaffinized with xylene, and rehydrated with graded ethanol. Antigen retrieval was performed with EDTA antigen retrieval buffer (pH 8.0) in a microwave oven. After blocking endogenous peroxides with 3% hydrogen peroxide solution, normal goat serum was incubated. Afterward, tissue slides were incubated overnight at 4 °C with specific primary antibodies. Following incubation with the secondary antibody, the tissue slides were incubated with horseradish peroxidase (HRP) conjugates (Zhongshan Goldenbridge, SP9001) with 3,3′-diaminobenzidine (DAB) staining for detection. Hematoxylin counterstaining was performed after the tissue sections were washed. Two researchers blinded to patients’ clinical information at different points in time randomly selected five fields of view to independently score the intensity of immunoreactivity under high magnification as 3 (strong), 2 (moderate), 1 (weak), and 0 (no staining), and the percentage of positively stained cells as 0 (less than 5%), 1 (5–25%), 2 (25–50%), 3 (50–75%), and 4 (> 75%). These two scores were multiplied to define the protein level of KIAA1429, and score 6 was used as the cutoff point to divide the specimens into “low” and “high” groups. An evaluation of the integrated optical density (IOD) of positive staining for KIAA1429 was performed by ImageJ software, and using IOD/area to calculate the mean IOD (MOD). Primary antibodies included KIAA1429 (Proteintech, 25712-1-AP), CHST11 (Invitrogen, PA5-103698), and Ki67 (Proteintech, 27309-1-AP).

### Western blotting analysis

Following the manufacturer’s instructions, protein extractions and western blotting were performed. Total protein from DLBCL cells was extracted by RIPA buffer (Pierce), 1× protease, and 1× phosphatase inhibitors (PhosSTOP, Roche, Basel, Switzerland). NE-PER Nuclear and Cytoplasmic Extraction Reagent (Thermo Fisher Scientific, MA, USA) was used to extract nuclear and cytoplasmic proteins. Protein concentrations were measured using a BCA Protein Detection Kit (Shenergy Biocolor). The samples were loaded onto SDS-PAGE gels for electrophoresis and then transferred to polyvinylidene fluoride membranes (Millipore, MA, USA) for immunoblotting. Afterward, the membranes were placed in a blocking solution (Tris-buffered saline containing 5% skim milk and 0.1% Tween 20) and incubated for 1 h, followed by overnight incubation at 4 °C with the indicated primary antibodies. After washing the membrane with TBS-T, the membrane was incubated with HRP-conjugated secondary antibody (Zhongshan Goldenbridge). Following treatment with a chemiluminescence detection reagent (Merck Millipore, MA, USA), the chemiluminescence signal was detected with an Amersham Imager 600 imaging system (General Electric, USA). Band quantification was performed using ImageJ software (NIH). The primary antibodies were against KIAA1429 (Proteintech, 25712-1-AP), CHST11 (Invitrogen, PA5-103698), Caspase-8 (Cell Signaling Technology (CST), 4790), Cleaved Caspase-8 (CST, 9748), Caspase-9 (CST, 9508), Cleaved Caspase-9 (CST, 92873), Caspase-3 (CST, 14220), Cleaved Caspase-3 (CST, 9664), PARP (CST, 9542), Cleaved PARP (CST, 5625), Bcl-2 (Abcam, ab32124), P21 (CST, 2947), Cyclin B1 (CST, 4138), MOB1B (ORIGENE, TA501388S), YAP (Proteintech,13584-1-AP), LATS1 (CST, 3477), Phospho-LATS1(T1079) (Proteintech, 28998-1-AP), Phospho-YAP(S127) (Abcam, ab76252), Smad2(phospho S467) (Abcam, ab280888), Smad3(phospho S423 + S425) (Abcam, ab52903), Smad2/3 (Abcam, ab202445), TEAD (Proteintech, 12418-1-AP), GAPDH (Zhongshan Goldenbridge, TA-08), and Histone H3 (CST, 4499).

### Bioinformatic analysis

On the basis of The Cancer Genome Atlas (TCGA) (https://portal.gdc.cancer.gov/) and the Gene Expression Omnibus (GEO) databases (https://www.ncbi.nlm.nih.gov/geo/) (GEO accession: GSE117556 and GSE56315), gene expression profiles were obtained. The clinicopathological information was downloaded from GSE117556 and a previous study [22]. Analysis of differentially expressed genes (DEGs) was conducted using the Limma package. Weighted gene co-expression network analysis (WGCNA) was carried out in R software using the “WGCNA” package. The adjacency matrix, topological overlap matrix (1-TOM), and dissimilarity (1-TOM) were constructed. Gene Ontology (GO) terms and Kyoto Encyclopedia of Genes and Genomes (KEGG) pathways were visualized using the “ggplot2” R package.

### In vivo subcutaneous xenograft model

We followed the guidelines of the Shandong Provincial Hospital Animal Care and Research Advisory Committee for all animal experiments. Twenty-eight 4-week-old female severe combined immunodeficiency (SCID) beige mice (*n* = 7 per group) were purchased from Beijing Vital River Laboratory Animal Technology Co., Ltd. (Beijing, China) and bred in pathogen-free conditions. A total of 1 × 10^7^ KIAA1429 stable knockdown OCI-LY1 cells or CHST11 stable knockdown OCI-LY1 cells were injected subcutaneously into the right armpit of mice. Two investigators who were blinded to the mice allocation observed the general condition of mice and tumor growth every 2 days, measuring tumor size with a vernier caliper upon the tumor size was higher than the skin surface and recording it. Tumor volume was calculated as *V* = (*a* × *b*^2^)/2, where *a* represents the largest dimension and *b* represents the vertical diameter. The tumor volume reached 2000 mm^3^, whereupon the mice were culled and the remaining mice were sacrificed after 4 weeks.

### Statistical analysis

Statistical analyses were conducted with R version 3.6.0, SPSS Statistics version 20.0, and GraphPad Prism 8.0. Overall survival (OS) was evaluated using the Kaplan–Meier method, and comparisons between groups were based on the log-rank test. The effect of selected variables on OS was determined through univariate and multivariate Cox regression analyses. Pearson’s correlation test was used for correlation analysis. Experimental data are presented as mean ± standard deviation (SD) based on three repetitions. Two-tailed Student’s *t*-tests were used to assess the significance of differences between the mean values between groups. An indication of statistical significance was *p* < 0.05.

## Results

### Integrated analysis of m6A regulators and identification of the prognostic value of KIAA1429 in DLBCL

**Fig. 1:**
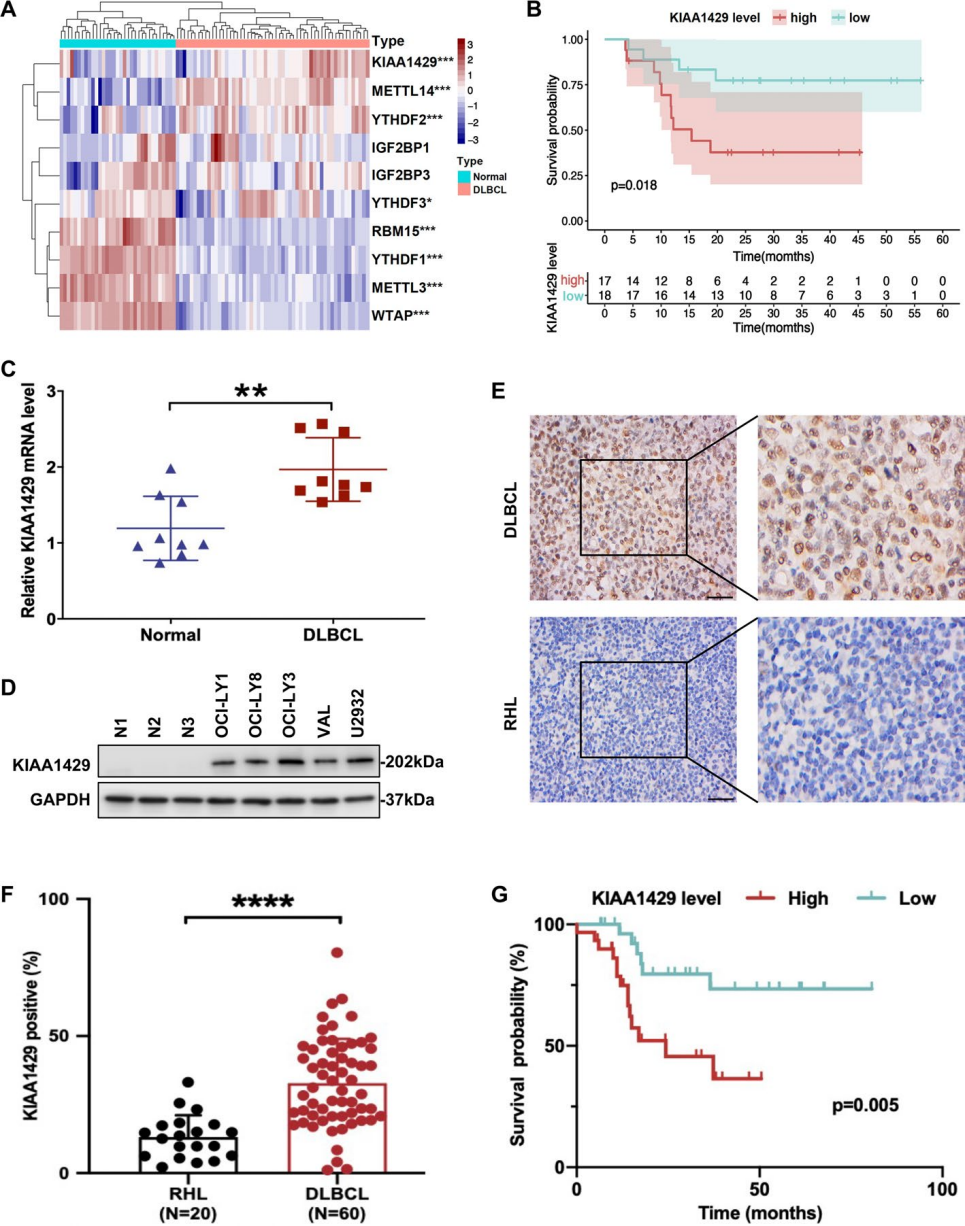
Dysregulated expression of m6A regulators and elevated KIAA1429 levels were correlated with disease progression in DLBCL.** A** Heatmap of m6A regulator expression in the healthy tonsil tissues (*n* = 33) and DLBCL tissues (*n* = 55) from the GEO database (GSE56315), with high and low expression levels shown in red and blue, respectively. **B** Survival curves indicated shorter survival times for patients with DHL with higher *KIAA1429* expression. **C** The mRNA level of *KIAA1429* in DLBCL cells (*n* = 3) was increased compared with that in CD19^+^ B cells (*n* = 3) as determined by RT-qPCR. Data are represented as mean ± SD of three independent experiments. **p < 0.01. **D** Western blotting indicated enhanced KIAA1429 expression in DLBCL cell lines. **E** Higher expression of KIAA1429 was found in DLBCL tissues (*n* = 60) than in RHL samples (*n* = 20). Bar, 50 μm. **F** Comparison of MOD values for KIAA1429-positive staining in DLBCL and RHL patients by two-tailed Student’s *t*-test. ****p < 0.0001. **G** Survival analysis revealed more prolonged survival times of patients with DLBCL with decreased KIAA1429 expression than those with increased KIAA1429 expression. The median overall survival was assessed by the log-rank test

To comprehensively dissect the contribution of m6A regulators in DLBCL, analyses by public databases from GEO (GSE56315) were performed, and the results unveiled differential expression of most of the m6A regulators (Fig. 1A). Subsequently, we speculated the variations in the ratio of m6A regulators as an intrinsic signature representing individual differences, and the results indeed showed that the proportion of differing m6A regulators ranged from weakly to strongly correlated in DLBCL (Additional file 2: Fig. S1A). To evaluate the prognostic significance of m6A regulators in DLBCL, 475 patients with GCB-DLBCL from GSE117556 were enrolled since it comprised all patients with double-hit lymphoma (DHL), who commonly presented poor prognosis in DLBCL [23]. Univariate Cox regression analysis revealed that six genes were at high risk and significantly associated with prognosis (Additional file 2: Fig. S1B). Additionally, LASSO Cox regression was performed to establish an m6A regulator-based risk model in DLBCL and calculate the risk score of patients with DLBCL (Additional file 2: Fig. S1C, D). On the basis of the median risk score, patients with DLBCL were classified into high-risk or low-risk groups, and significantly shorter survival times were found in the high-risk group (*p* < 0.001; Additional file 2: Fig. S1E). Moreover, ROC curves with an AUC value of 0.706 validated the reliability of the risk model in prognostic prediction of DLBCL (Additional file 2: Fig. S1F). Univariate and multivariate Cox regression analyses unveiled the risk score as an independent prognostic indicator in patients with DLBCL (Additional file 2: Fig. S1G, H). Together, these data suggested that many m6A regulators were differentially expressed and correlated with poor prognosis in DLBCL.

To further identify a robust prognostic biomarker based on m6A regulators in patients with DLBCL, the relationship between prognosis-related m6A regulators and the clinicopathological features of patients with DHL was evaluated. The results indicated that KIAA1429 was correlated with the high International Prognostic Index (IPI) score and DHL, while the correlation of the others was not remarkably dissimilar (*p* < 0.05; Additional file 2: Fig. S1I, J and Additional file 1: Table S3). Furthermore, survival analysis showed better OS in patients with DHL with decreased *KIAA1429* expression (*p* = 0.018; Fig. 1B), whereas *KIAA1429* expression was not statistically significant for OS in non-DHL patients (Additional file 2: Fig. S1K). Univariate Cox regression analysis unveiled that age, stage, Eastern Cooperative oncology Group  (ECOG) score, lactate dehydrogenase (LDH), IPI score, and *KIAA1429* expression were substantially related to 5-year OS in patients with GCB-DLBCL (Additional file 2: Fig. S1L).

We next verified the expression of KIAA1429 in DLBCL, finding higher levels of KIAA1429 mRNA and protein in DLBCL cells than in normal B lymphocytes (*p* < 0.01; Fig. 1C, D). Elevated KIAA1429 protein levels were detected in 83.3% (50/60) of DLBCL tissues and 15% (3/20) of RHL tissues by IHC staining (*p* < 0.05; Fig. 1E, F). The clinical implications of KIAA1429 upregulation in patients with DLBCL were further validated in our center. Increased expression of KIAA1429 was associated with DLBCL subtypes (*p* = 0.034) and elevated Ann Arbor stage (*p* = 0.007) (Table 1), indicating a positive correlation between high KIAA1429 expression and DLBCL disease progression. Additionally, survival analysis of patients with DLBCL presented shorter survival times in patients with elevated KIAA1429 expression (*p* = 0.005; Fig. 1G). Thus, these results revealed that KIAA1429 was highly expressed and might be an independent prognostic indicator for patients with DLBCL.

**Table 1:** Correlation between KIAA1429 expression and clinical features of patients with DLBCL

Clinical variables	No. of patients	KIAA1429 expression		*p* value
		Positive	Negative	
Subtype				
GCB	24	17 (70.8%)	7 (29.1%)	0.034*
Non-GCB	36	33 (91.7%)	3 (8.3%)	
Age (years)				
< 60	28	24 (85.7%)	4 (14.3%)	0.643
≥ 60	32	26 (81.3%)	6 (18.7%)	
Gender				
Male	36	31 (86.1%)	5 (13.9%)	0.48
Female	24	19 (79.2%)	5 (20.8%)	
Ann Arbor stage				
I or II	20	13 (65%)	7 (35%)	0.007**
III or IV	40	37 (92.5%)	3 (7.5%)	
B symptoms				
Present	10	9 (90%)	1 (10%)	0.535
Absent	50	41 (82%)	9 (18%)	
Serum LDH				
Normal	31	23 (72.2%)	8 (27.8%)	0.05
Elevated	29	27 (93.1%)	2 (6.9%)	
Extranodal involvement				
Present	55	46 (83.6%)	9 (16.4%)	0.853
Absent	5	4 (80%)	1 (20%)	
IPI score				
0–2	27	20 (74.1%)	7 (25.9%)	0.082
3–5	33	30 (90.9%)	3 (9.1%)	

The bold values mean statistically significant

*GCB* germinal center B cell-like, *LDH* lactate dehydrogenase, *IPI* International Prognostic Index

*p < 0.05, **p < 0.01

### KIAA1429 facilitated cell proliferation, inhibited apoptosis, and promoted cell cycle progression in DLBCL

**Fig. 2:**
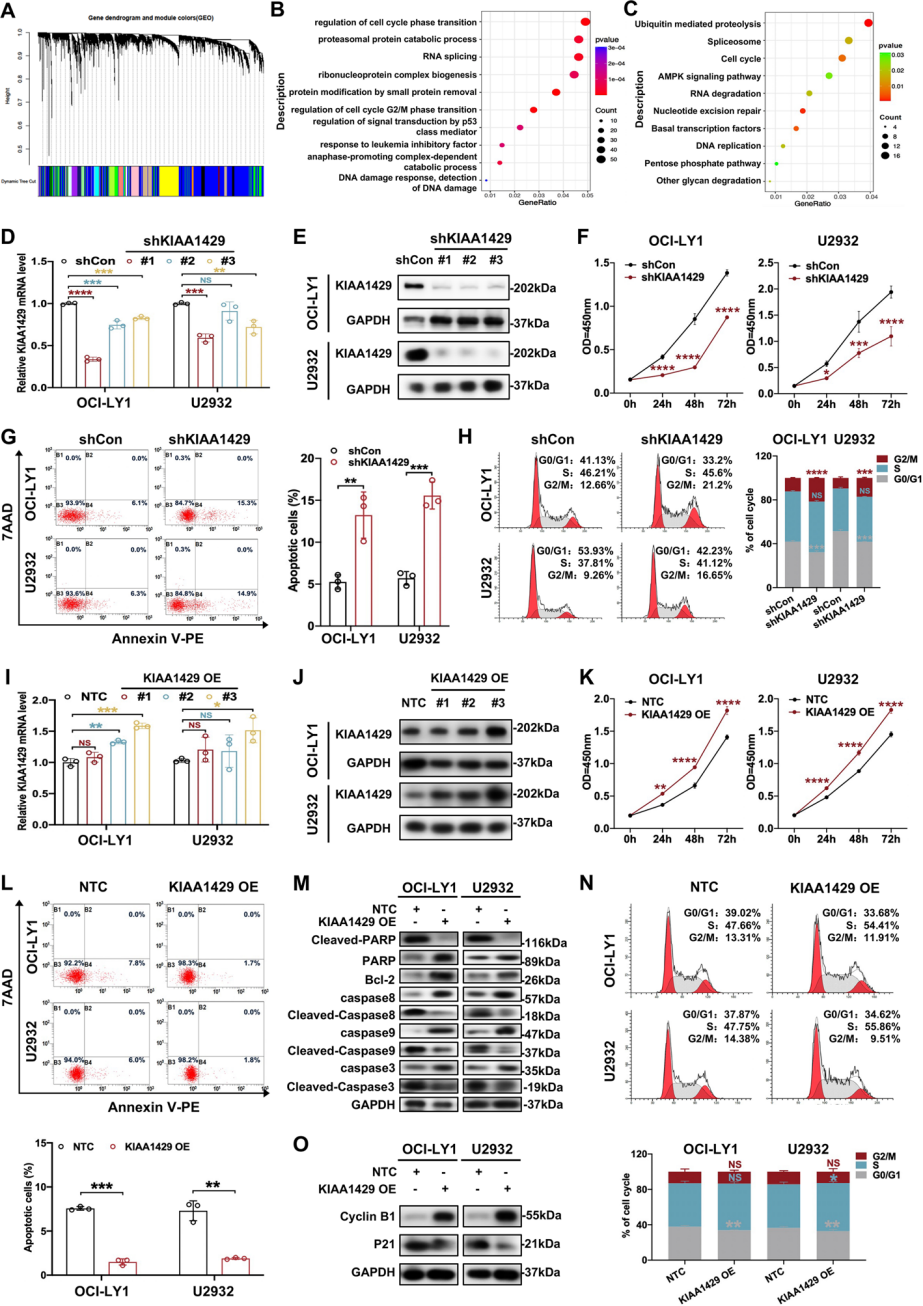
KIAA1429 promoted cell proliferation, inhibited apoptosis, and facilitated cell cycle progression in DLBCL. **A** Dendrogram showing hierarchical clustering of genes in distinct modules. **B**, **C** GO and KEGG analyses of genes correlated with KIAA1429 in DLBCL according to the GEO database (GSE117556). **D**, **E** RT-qPCR and western blotting analyses verified the knockdown effect of KIAA1429 at the mRNA and protein levels, respectively. At least three independent experiments were conducted to obtain the data presented as mean ± SD. **p < 0.01; ***p < 0.001; ****p < 0.0001. **F** Knockdown of KIAA1429 inhibited DLBCL cell proliferation as determined by CCK-8 assay. Triplicate data are presented as mean ± SD. *p < 0.05; ***p < 0.001; ****p < 0.0001. **G** Cell apoptosis was suppressed by knockdown of KIAA1429. Left: representative results; Right: statistical data of three independent experiments are shown as mean ± SD. **p < 0.01. **H** Induction of cell cycle arrest in G2/M phase upon KIAA1429 knockdown detected by flow cytometry. Left: representative results; Right: as a result of three independent experiments, mean ± SD are shown. ***p < 0.001; ****p < 0.0001. **I**, **J** Endogenous KIAA1429 overexpression by the CRISPR/dCas9-VP64 gene activation system was demonstrated at both the mRNA and protein levels. (NTC represents nontarget control; KIAA1429 OE represents overexpression of KIAA1429). Mean ± SD is shown for three independent experiments. *p < 0.05; **p < 0.01; ***p < 0.001. **K** The cell proliferation rate was enhanced by overexpression of KIAA1429 as detected by CCK8 assay. An average of three independent experiments is represented as mean ± SD. **p < 0.01; ****p < 0.0001. **L**, **M** Overexpression of KIAA1429 reduced cell apoptosis and modulated the expression of apoptosis-related proteins. Top: representative results; bottom: data from three independent experiments are presented as mean ± SD. **p < 0.01. **N**, **O** Cell cycle distribution and the regulation of cell cycle-related proteins upon KIAA1429 overexpression are displayed. Top: representative results; bottom: data are represented as mean ± SD. *p < 0.05; **p < 0.01

To explore the functional role of KIAA1429 in DLBCL, WGCNA was first performed to cluster genes into distinct modules based on the similarity of gene expression patterns (GSE117556). Using the dynamic tree cut, 26 gene coexpression modules were identified (Fig. 2A, Additional file 3: Fig. S2A). The top 40 genes associated with *KIAA1429* are shown in the heatmap (Additional file 3: Fig. S2B). Since genes in coexpressed modules generally share similar biological functions, the blue module, including *KIAA1429*, was further analyzed. GO analysis showed that KIAA1429 functioned in cell proliferation, cell cycle, apoptosis, etc. (Fig. 2B). KEGG analysis indicated that KIAA1429 was involved in cancer-related pathways, including MAPK signaling pathway and pentose phosphate pathway (Fig. 2C). We further conducted KEGG enrichment analysis with GSE117556 database to detect the associated genes and pathways with *KIAA1429* expression elevation. As is shown in Additional file 1: Table S4, pentose phosphate pathway, valine, leucine and isoleucine degradation, glutathione metabolism, and DNA replication were significantly enriched.

Loss- and gain-of-function assays were performed further to validate the biological function of KIAA1429 in DLBCL cells. Effective knockdown of KIAA1429 at both mRNA and protein levels was confirmed in DLBCL cells, with shKIAA1429#1 showing higher efficacy in OCI-LY1 and U2932 cells (Fig. 2D, E). DLBCL cell proliferation was diminished upon knockdown of KIAA1429 (Fig. 2F**)**. Moreover, KIAA1429 knockdown was found to increase cell apoptosis rates (Fig. 2G). Further analysis showed that KIAA1429 knockdown enhanced the fraction of cells in the G2/M phase (Fig. 2H). Similarly, overexpression of endogenous KIAA1429 was validated at both mRNA and protein levels (Fig. 2I, J). As expected, overexpression of KIAA1429 facilitated proliferation (Fig. 2K) and suppressed apoptosis of DLBCL cells (Fig. 2L). The expression of antiapoptotic protein Bcl-2 was increased while expression levels of proapoptotic proteins, including Cleaved-Caspase 3, Cleaved-Caspase 8, Cleaved-Caspase 9 and Cleaved-PARP, were decreased in DLBCL cells overexpressing KIAA1429 (Fig. 2M). Furthermore, overexpression of KIAA1429 was not accompanied by cell cycle arrest (Fig. 2N). Decreased expression of cell-cycle inhibitor P21 was discovered, and Cyclin B1, a marker of late G2 and mitosis (G2/M), was elevated (Fig. 2O). Collectively, these findings revealed that KIAA1429 might promote DLBCL progression by modulating proliferation, apoptosis, and cell cycle.

### Targeted deletion of KIAA1429 inhibited DLBCL tumor growth both in vitro and in vivo

**Fig. 3:**
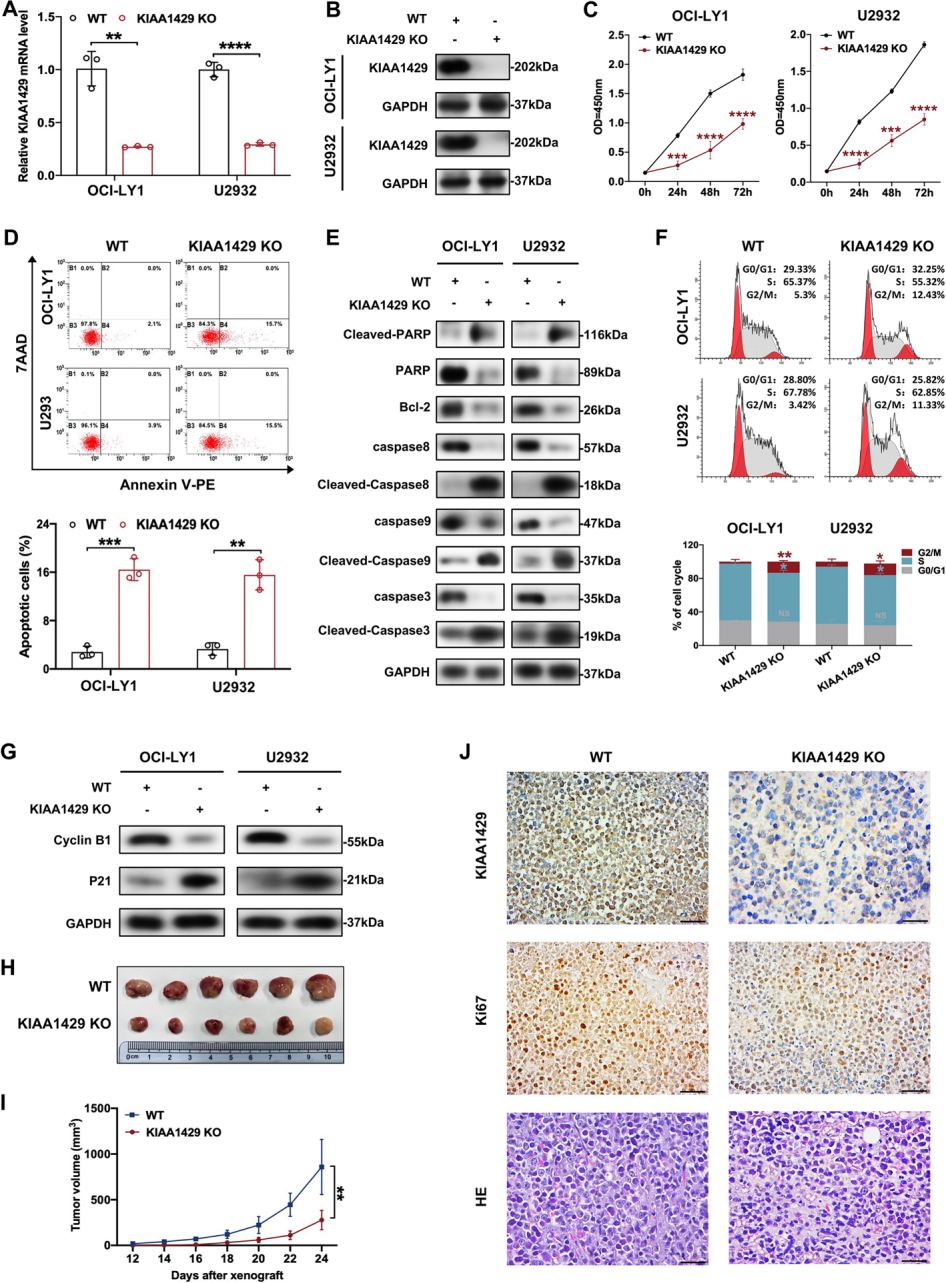
Deletion of KIAA1429 in DLBCL cells exerted antitumor effects both in vitro and in vivo. **A**, **B** Stable knockout of KIAA1429 in DLBCL cells by CRISPR/Cas9 was confirmed by RT-qPCR and western blotting. (WT represents wild-type cells; KIAA1429 KO represents CRISPR/CAS9 knockout cells). Three independent experiments were performed to obtain the data presented as mean ± SD. **p < 0.01; ****p < 0.0001. **C** Knockout of KIAA1429 dramatically suppressed the proliferation of DLBCL cells as determined by CCK8 assay. Three independent experiments are presented as mean ± SD. ***p < 0.001; ****p < 0.0001. **D**, **E** KIAA1429 deletion facilitated apoptosis and affected the expression of apoptosis-related proteins. Top: representative results; bottom: data from three independent experiments are represented as mean ± SD. **p < 0.01; ***p < 0.001. **F**, **G** The cell cycle was arrested in G2/M phase followed by alterations in cell cycle-associated proteins upon KIAA1429 knockout. Top: representative results; bottom: statistical results from three independent experiments are represented as mean ± SD. *p < 0.05; **p < 0.01. **H**, **I** SCID beige mice were subcutaneously injected with OCI-LY1 cells with or without KIAA1429 deletion (*n* = 6), and tumor sizes and volumes were decreased in KIAA1429 knockout cells. **p < 0.01. **J** Histological analysis of excised tumors by hematoxylin/eosin (HE) staining and Ki67. Bar, 50 μm

To further consolidate our findings above, we targeted KIAA1429 for deletion in DLBCL cells by CRISPR/Cas9 technology (Fig. 3A, B). As observed in knockdown models, knockout of KIAA1429 significantly inhibited DLBCL cell proliferation (Fig. 3C). Flow cytometry demonstrated that deletion of KIAA1429 markedly enhanced cell apoptosis rates (Fig. 3D). After deletion of KIAA1429, antiapoptotic protein Bcl-2 was appreciably decreased, and the levels of proapoptotic proteins (Cleaved-Caspase 3, Cleaved-Caspase 8, Cleaved-Caspase 9, and Cleaved-PARP) were significantly enhanced (Fig. 3E). Moreover, the proportion of DLBCL cells in the G2/M phase was remarkably elevated upon KIAA1429 deletion (Fig. 3F). Notably, higher levels of P21 expression and lower levels of Cyclin B1 were observed in DLBCL cells with KIAA1429 knockout (Fig. 3G). To further address the contribution of KIAA1429 deletion on tumorigenicity in vivo, subcutaneous xenograft mouse models were established (*n* = 6 per group). By comparing tumor growth curves and tumor volumes between the knockout and control groups, we found that KIAA1429 deletion markedly decreased tumor growth in vivo (Fig. 3H, I). IHC staining of proliferation-related protein Ki67 indicated that cell proliferation was appreciably suppressed upon KIAA1429 knockout (Fig. 3J). These findings demonstrated an antitumor effect in vitro and in vivo with KIAA1429 knockout in DLBCL cells.

### Identification of CHST11 as a downstream target of KIAA1429-mediated m6A modification

**Fig. 4:**
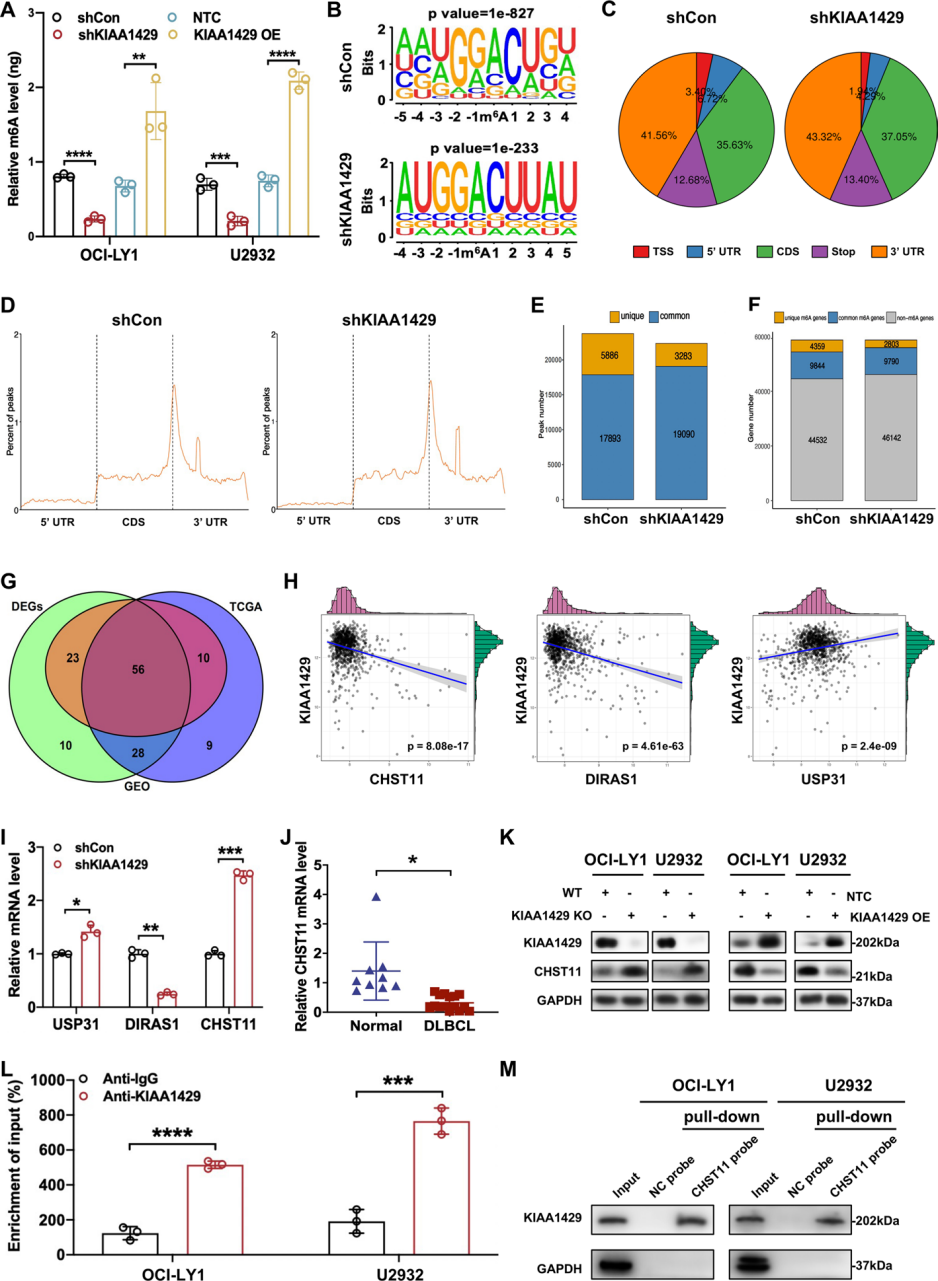
CHST11 was identified as a downstream target of KIAA1429-mediated m6A modification on DLBCL. **A** A colorimetric quantification assay was used to detect the m6A levels of total RNA in OCI-LY1 and U2932 cells with KIAA1429 knockdown or overexpression. Triplicate data are presented as mean ± SD. ***p < 0.001; ****p < 0.0001. **B** HOMER with MeRIP-seq identified the top consensus motif of m6A peaks in OCI-LY1 cells with or without knockdown of KIAA1429. **C**, **D** The percentage of total m6A peak distribution in the specified regions was determined in cells with control and KIAA1429 knockdown. **E** The number of m6A peaks in control and KIAA1429-deficient cells was determined by MeRIP-seq. **F** The number of m6A-modified genes was revealed by MeRIP-seq, where at least one common m6A was present in the common m6A genes, whereas unique m6A genes did not contain common m6A peaks. **G** Venn diagram exhibiting overlap among differentially expressed genes (DEGs) from MeRIP-seq and RNA-seq and genes associated with KIAA1429 from TCGA and GEO databases. **H** Pearson correlations between expression of *KIAA1429* and the top three genes. **I** RT-qPCR was used to detect the mRNA levels of the top three genes in cells with or without KIAA1429 knockdown. Results are expressed as mean ± SD from three independent experiments. *p < 0.05; **p < 0.01; ***p < 0.001. **J** A diminished level of *CHST11* in DLBCL cells was found by RT-qPCR, compared with normal B cells. Statistical analyses are based on at least three independent experiments with mean ± SD. *p < 0.05. **K** The protein expression of KIAA1429 and CHST11 was negatively correlated as confirmed by western blotting. **L** RIP-qPCR showed direct binding between KIAA1429 and *CHST11* mRNA in DLBCL cells. Data are represented as mean ± SD from three independent experiments. ***p < 0.001; ****p < 0.0001. **M** In vitro binding of *CHST11* probes with KIAA1429 protein was demonstrated by RNA pulldown followed by western blotting

Since KIAA1429 is an m6A methyltransferase [24], we explored the regulatory effect of KIAA1429 on m6A levels in DLBCL cells. As expected, KIAA1429 knockdown remarkably reduced the global m6A levels of DLBCL cells, whereas KIAA1429 overexpression elevated it (Fig. 4A). To dissect the involved mechanism of KIAA1429 function in DLBCL progression, RNA-seq and MeRIP-seq in DLBCL cells with stable KIAA1429 knockdown were performed. RNA-seq showed the differential expression of 609 genes after KIAA1429 knockdown, of which 336 were downregulated and 273 were upregulated (Additional file 4: Fig. S3A). MeRIP-seq indicated that m6A peaks of 9806 transcripts presented differential abundance, with 1276 upregulated and 8530 downregulated (Additional file 4: Fig. S3B). Consistent with previous studies [25, 26], our MeRIP-seq revealed that both control and KIAA1429 knockdown cells exhibited high levels of consensus m6A sequence “RRACH” (R = G or A; H = A, C, or U) (Fig. 4B). Moreover, pie charts and metagene analysis of m6A-peak distribution showed that m6A peaks were most prevalent in coding sequences (CDSs) and 3′ untranslated regions (UTRs) of mRNA, with the highest enrichment surrounding the 3′ UTR (Fig. 4C, D).

To further identify the target transcripts of KIAA1429, the differential peaks from MeRIP-seq were further analyzed. In control and KIAA1429 knockdown cells, 23,779 and 22,273 m6A peaks were identified from 14,243 and 12,593 m6A-modified transcripts, respectively (Fig. 4E). A total of 5886 peaks disappeared, 3283 new peaks appeared in DLBCL cells with KIAA1429 knockdown, and the other 19,090 peaks were observed in both control and knockdown cells (Fig. 4E). Given that KIAA1429 is an m6A methyltransferase, we speculated that genuine targets might be contained in the unique peaks of 5886 with 4359 unique genes (Fig. 4F). The correlation between these peaks and DEGs obtained from our RNA-seq was explored. A total of 135 genes were identified by filtering the 5886 unique m6A peaks with the 437 DEGs whose expression changed by at least twofold (Additional file 4: Fig. S3C). Subsequently, correlation analysis between KIAA1429 expression and the above genes was performed on samples from patients with DLBCL in TCGA and GEO (GSE117556) datasets, finding remarkable associations between expression of KIAA1429 and 56 genes (Fig. 4G). The top correlated genes, carbohydrate sulfotransferase 11 (*CHST11*), ubiquitin-specific peptidase 31 (*USP31*), and DIRAS family GTPase 1 (*DIRAS1*), were further visualized, where *KIAA1429* expression was positively associated with *DIRAS1* and negatively correlated with *CHST11* and *USP31* (Fig. 4H). We verified these results by RT-qPCR, and the mRNA levels of *CHST11* were found to be remarkably elevated upon KIAA1429 knockdown compared with the other two genes (Fig. 4I). Interestingly, the mRNA level of *CHST11* was notably decreased in DLBCL cells (Fig. 4J), whereas the expression of *USP31* and *DIRAS1* was not remarkably distinct between DLBCL cells and normal B cells (Additional file 4: Fig. S3D, E), indicating that CHST11 may contribute most to the regulation of DLBCL progression. Moreover, knockout of KIAA1429 enhanced the protein level of CHST11, whereas overexpression of KIAA1429 reduced it, suggesting a regulatory relationship of KIAA1429 on CHST11 expression (Fig. 4K). RIP with an antibody against KIAA1429 followed by RT-qPCR indicated that KIAA1429 bound to *CHST11* mRNA (Fig. 4L). The direct binding interaction between *CHST11* RNA and KIAA1429 was further validated by immunoblotting after pulldown of biotin-labeled *CHST11* RNA on KIAA1429 protein (Fig. 4M). Therefore, our results revealed that KIAA1429 modulated the mRNA and protein levels of CHST11 in DLBCL, which was identified as its direct target.

### KIAA1429 reduced the stability of *CHST11* mRNA via an m6A-YTHDF2-dependent mechanism

**Fig. 5:**
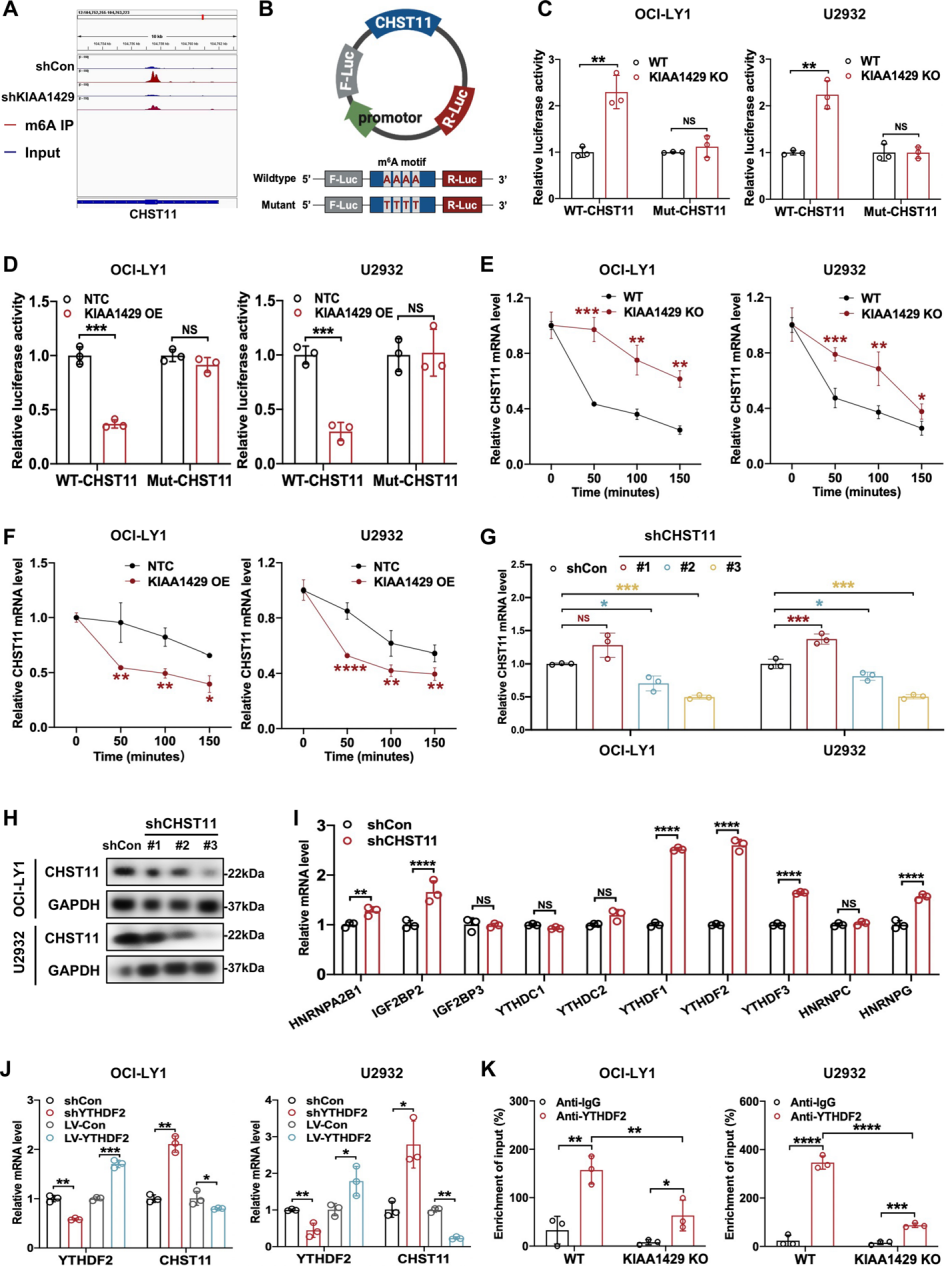
KIAA1429 reduced the stability of *CHST11* mRNA in an m6A-YTHDF2-dependent manner. **A** Visualization of m6A peak in *CHST11* transcripts based on MeRIP-seq, and reduction of m6A levels of *CHST11* by knockdown of KIAA1429. **B** Construction of a luciferase reporter containing wild-type *CHST11* 3′ UTR and *CHST11* 3′ UTR with a mutation at the m6A consensus sequence. **C**, **D** An analysis of relative luciferase activity was conducted in DLBCL cells with or without KIAA1429 knockdown or KIAA1429 overexpression. Triplicate data are shown as mean ± SD. **p < 0.01; ***p < 0.001. **E**, **F** RT-qPCR was used to detect the expression of *CHST11* after treatment with actinomycin D (5 μg/ml) at the indicated timepoints in KIAA1429 knockdown, KIAA1429-overexpressing, and corresponding control DLBCL cells. Result are presented as mean ± SD from three independent experiments. *p < 0.05; **p < 0.01; ***p < 0.001; ****p < 0.0001. **G**, **H** Knockdown efficiency of CHST11 in DLBCL cells was verified at mRNA and protein levels. As a result of three independent experiments, mean ± SD is presented. *p < 0.05; ***p < 0.001. **I** RT-qPCR was utilized to determine the alterations in expression of m6A-binding proteins upon CHST11 knockdown. The results are shown as mean ± SD from three independent experiments. **p < 0.01; ****p < 0.0001. **J** The relative mRNA levels of *CHST11* were detected in DLBCL cells with YTHDF2 overexpression or knockdown. Data from three independent experiments are presented as mean ± SD. *p < 0.05; **p < 0.01; ***p < 0.001. **K** Validation of direct binding between YTHDF2 and *CHST11* mRNA was performed by RIP-qPCR. Data are expressed as mean ± SD from three independent experiments. **p < 0.01; ***p < 0.001; ****p < 0.0001

KIAA1429 was found to target the 3′ UTR of *CHST11* mRNA, which presented a statistically diminished m6A peak in KIAA1429 knockdown cells compared with control (Fig. 5A). This result prompted us to speculate that KIAA1429 directly regulates the m6A modification in 3′ UTR of *CHST11* mRNA, thereby interfering with its expression. Subsequently, luciferase reporters harboring either the wild-type or mutant *CHST11* 3′ UTR was constructed, where the adenosine in the mutant reporter was substituted by thymine to abolish m6A modification (Fig. 5B). As expected, a luciferase reporter containing wild-type *CHST11* 3′ UTR upon KIAA1429 deletion exhibited a clear increase in luciferase activity, whereas mutation at the m6A sites abrogated this enhancement (Fig. 5C). Conversely, overexpression of KIAA1429 diminished the luciferase activity of wild-type *CHST11* 3′ UTR but failed to interfere with the expression of the mutant *CHST11*-fused reporter, indicating that m6A modification mediated by KIAA1429 regulated CHST11 expression (Fig. 5D). To address whether KIAA1429 modulated the stability of *CHST11* mRNA, we treated DLBCL cells with KIAA1429 knockdown or overexpression with actinomycin D at indicated timepoints. A significant increase in *CHST11* mRNA stability was found in KIAA1429 knockout cells in the presence of actinomycin D, while KIAA1429 overexpression decreased it, revealing that KIAA1429 was necessary for decreasing the stabilization of *CHST11* (Fig. 5E, F).

The readers of m6A are considered essential to function in modulating gene expression [27, 28]. To explore how m6A modification regulates CHST11 expression, the interaction between CHST11 and m6A binding proteins, including YTH domain family (YTHDF) proteins, IGF2BPs, and hnRNP proteins, was analyzed. RNA interference experiments targeting CHST11 were conducted in DLBCL cells, with shCHST11#3 presenting higher efficiency at mRNA and protein levels (Fig. 5G, H). Interestingly, after knockdown of CHST11, YTHDF2 was most significantly differentially expressed compared with other binding proteins (Fig. 5I). Moreover, the transcript level of *CHST11* was markedly increased in YTHDF2 knockdown cells but reduced in YTHDF2-overexpressing cells (Fig. 5J). Furthermore, RIP analysis with an antibody against YTHDF2 followed by RT-qPCR suggested that YTHDF2 bound to *CHST11* mRNA, and the amount of binding was decreased by KIAA1429 deletion (Fig. 5K). Collectively, these results revealed that KIAA1429-mediated m6A modification in DLBCL cells diminished the stability of *CHST11* mRNA and suppressed its expression in a YTHDF2-dependent manner.

### CHST11 knockdown exerted tumor-promoting effects and abolished tumor suppression induced by KIAA1429 knockdown

**Fig. 6:**
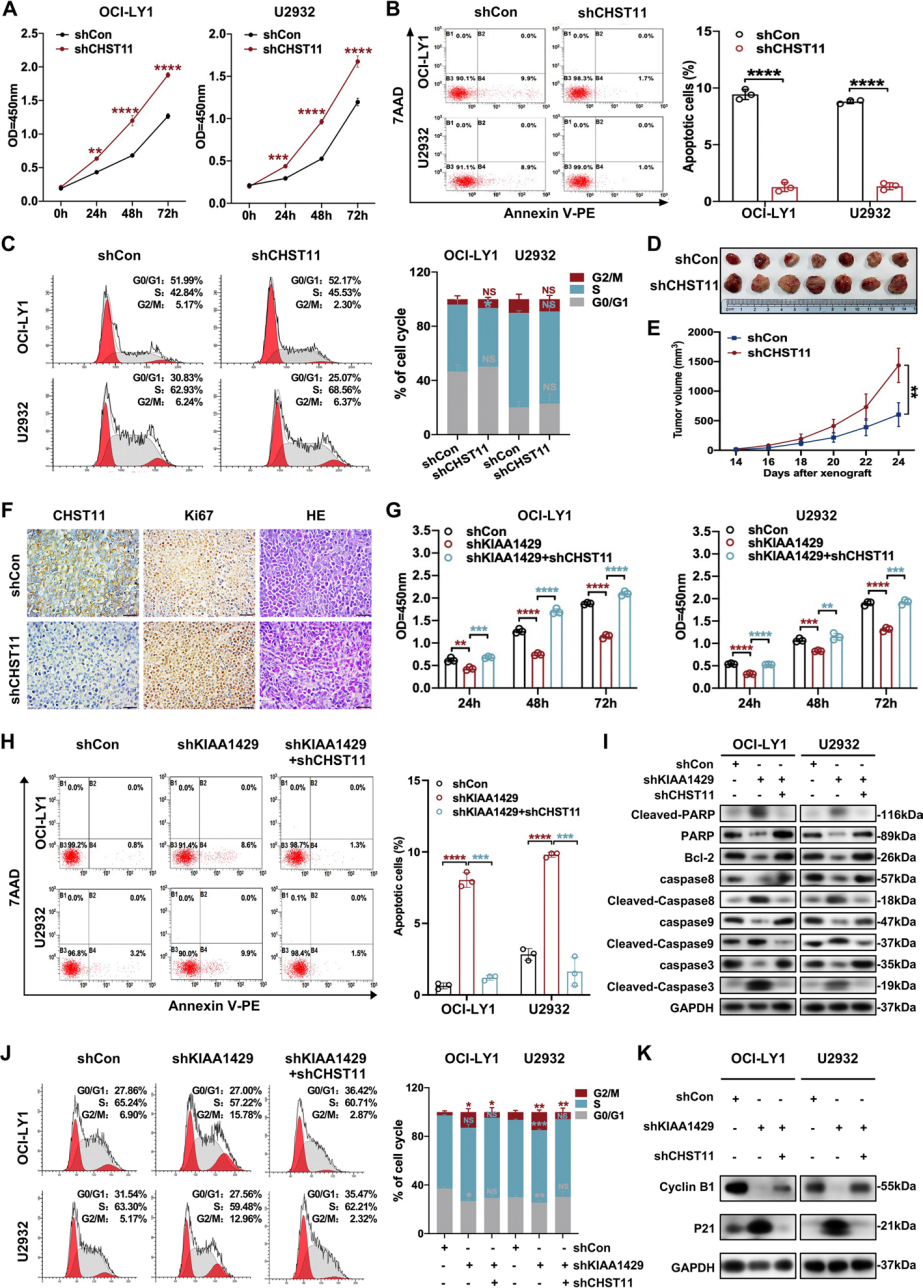
CHST11 functioned as a tumor suppressor in DLBCL. **A** Knockdown of CHST11 promoted cell proliferation in DLBCL. Mean ± SD is presented for three independent experiments. **p < 0.01; ***p < 0.001; ****p < 0.0001. **B** The apoptosis rate was reduced in CHST11 knockdown cells compared to control cells. Left: representative results; right: statistical data are presented as mean ± SD based on three independent experiments. ****p < 0.0001. **C** Cell cycle distribution of control and CHST11-deficient DLBCL cells was determined by flow cytometry. Left: representative results; right: data are expressed as mean ± SD from three independent experiments. *p < 0.05. **D**, **E** OCI-LY1 cells with or without CHST11 depletion were injected subcutaneously into SCID beige mice (*n* = 7), and CHST11 knockdown cells presented increased tumor size and tumor volume. **F** Histological analysis of resected tumors was performed by hematoxylin/eosin (HE) staining and Ki67 staining. Bar, 50 μm. **G** Cell proliferation was assessed in DLBCL cells with or without KIAA1429 knockdown or combined KIAA1429 knockdown and CHST11 knockdown. Data from three independent experiments are presented as mean ± SD. **p < 0.01; ***p < 0.001; ****p < 0.0001. **H**, **I** Examination of apoptosis and the expression of apoptosis-related proteins in DLBCL cells with or without KIAA1429 knockdown or in combination with KIAA1429 knockdown and CHST11 knockdown. Left: representative results; Right: data from three independent experiments are represented as mean ± SD. ***p < 0.001; ****p < 0.0001. **J**, **K** Flow cytometry was used to detect the distribution of cell cycle in DLBCL cells with or without KIAA1429 knockdown or combined KIAA1429 knockdown and CHST11 knockdown. Left: representative results; Right: data are shown as mean ± SD from three independent experiments. *p < 0.05; **p < 0.01; ***p < 0.001

CHST11, a member of the HNK1 family of Golgi-associated sulfotransferases, is primarily expressed in the hematopoietic cells [29, 30]. The expression of CHST11 in B-cell chronic lymphocytic leukemia was reported to be dysregulated [30]. We next explored the functional involvement of CHST11 in DLBCL. Enhanced cell proliferation rates and decreased cell apoptosis were found in stable CHST11 knockdown cells (Fig. 6A, B). In addition, the distribution of cell-cycle phases exhibited no significant alteration upon CHST11 knockdown (Fig. 6C). We next constructed xenograft mouse models to evaluate the functional role of CHST11 in DLBCL in vivo (*n* = 7 per group). Knockdown of CHST11 resulted in remarkable enhancement in tumor volumes and acceleration of tumor growth rates (Fig. 6D, E). Moreover, the expression of Ki67 was elevated in CHST11-deficient cells, further suggesting CHST11 as a tumor suppressor in DLBCL (Fig. 6F).

Since KIAA1429 induced downregulation of CHST11 expression, we explored whether this regulation involved the tumor-promoting role of KIAA1429 in DLBCL. Consistent with expectations, the reduction in cell proliferation and increase in apoptosis caused by KIAA1429 knockdown were considerably abrogated by CHST11 depletion in these cells (Fig. 6G, H). KIAA1429 knockdown-elicited elevated expression of cleaved pro-apoptotic proteins and reduced anti-apoptotic proteins were reversed by CHST11 depletion (Fig. 6I). Notably, knockdown of CHST11 diminished the phenotype of G2/M phase cell cycle arrest caused by KIAA1429 depletion (Fig. 6J). Similarly, KIAA1429 depletion elicited high P21 expression and low Cyclin B1 expression, which was reversed by knockdown of CHST11 (Fig. 6K). Together, these data revealed that CHST11 suppressed tumor growth, and KIAA1429 exerted a tumor-promoting effect by inhibiting expression of CHST11 in DLBCL.

### KIAA1429 regulated Hippo–YAP signaling by interacting with CHST11

**Fig. 7:**
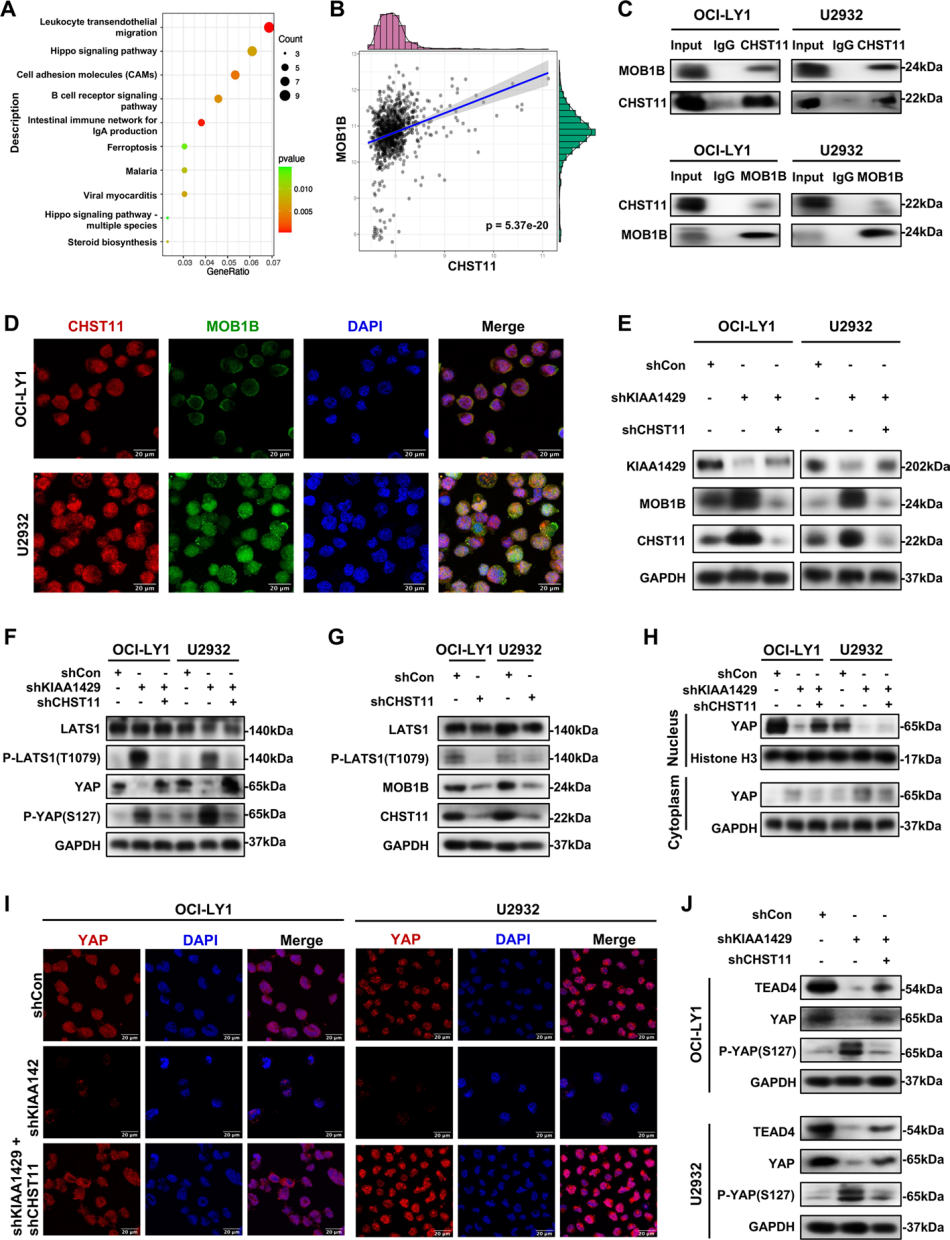
KIAA1429 inactivated Hippo–YAP signaling by interacting with CHST11. **A** GO enrichment analysis showed high enrichment of the Hippo signaling pathway based on RNA-seq. **B** A positive correlation between *CHST11* and *MOB1B* was found in Pearson correlation analysis. **C** The binding between CHST11 and MOB1B proteins was confirmed by co-IP in OCI-LY1 and U2932 cells. **D** Colocalization of CHST11 and MOB1B protein in OCI-LY1 and U2932 cells was exhibited in confocal immunofluorescent images. Bar, 20 μm. **E** The protein level of MOB1B was detected in DLBCL cells with or without KIAA1429 knockdown or with combined KIAA1429 knockdown and CHST11 knockdown. **F** Expression of core proteins in the Hippo pathway was examined in DLBCL cells with or without KIAA1429 knockdown or with combined KIAA1429 knockdown and CHST11 knockdown. **G** Knockdown of CHST11 suppressed MOB1B protein expression and LATS1 phosphorylation. **H** YAP expression was detected by western blotting after isolation and extraction of cytoplasmic and nuclear proteins. **I** Endogenous expression and subcellular localization of YAP were assessed by immunofluorescence staining. Bar = 20 μm. Data were obtained from three independent experiments. **J** Western blotting showed that knockdown of CHST11 rescued the KIAA1429 knockdown-induced decrease in YAP and TEAD4 expression and increase in YAP phosphorylation

We next further analyzed the data from our RNA-seq and MeRIP-seq to gain mechanistic insight into how KIAA1429 functioned in DLBCL progression following induction of CHST11 downregulation. GO and KEGG enrichment analyses based on MeRIP-seq indicated that gene expression, cell cycle, apoptosis, and metabolic pathways were enriched (Additional file 5: Fig. S4A, B). KEGG enrichment analysis by RNA-seq showed significant enrichment of DEGs in the Hippo signaling pathway (Fig. 7A). The Hippo pathway is crucial for controlling organ size and suppressing tumor growth, and accumulating evidence also suggests its involvement in metabolic regulation [31, 32]. To determine whether KIAA1429 acted as a regulator in Hippo–YAP signaling by modulating CHST11, we analyzed the correlation between CHST11 and Hippo–YAP pathway core components using data from GSE117556. The related genes including *MOB1B*, *NF2*, *LATS2*, *LATS1*, and *MOB1A* are shown (*p* < 0.05, Additional file 5: Fig. S4C). Among them, MOB1B, a core kinase of the Hippo–YAP pathway, ranked first and was positively correlated with *CHST11* (*p* < 0.0001, Fig. 7B). Physical interaction between endogenous CHST11 protein and MOB1B protein was determined by coimmunoprecipitation (co-IP) and western blotting (Fig. 7C). In addition, immunofluorescent confocal imaging analysis of OCI-LY1 and U2932 cells revealed colocalization of CHST11 protein and MOB1B protein (Fig. 7D).

Furthermore, the expression of Hippo–YAP pathway core components correlated with CHST11, including MOB1B, LATS1, phosphorylated (p) LATS1, YAP, and P-YAP serine 127 [P-YAP (S127)], was detected in DLBCL cells with KIAA1429 knockdown alone or simultaneous knockdown of KIAA1429 and CHST11. We found that knockdown of KIAA1429 enhanced CHST11 protein expression and resulted in elevated expression of MOB1B protein (Fig. 7E). The phosphorylation of Hippo–YAP pathway core components, including LATS1 and YAP (S127), was increased upon KIAA1429 knockdown, suggesting activation of Hippo–YAP signaling, while it was abrogated by CHST11 depletion in these cells (Fig. 7F). Interestingly, total YAP was diminished in KIAA1429-deficient cells, which was reversed by knockdown of CHST11, yet total LATS1 was unaffected in these cells (Fig. 7F).

Activation of Hippo signaling results in phosphorylation of YAP at the S127 site by LATS1 to promote its cytoplasmic translocation and subsequent degradation. Moreover, MOB1B is required for the complete activation of LATS1 [33]. These findings prompted us to verify whether LATS1 was activated by CHST11 binding to MOB1B and whether KIAA1429 through regulation of CHST11 expression altered YAP activity to modulate the Hippo–YAP signaling. We found that MOB1B expression and LATS1 phosphorylation were decreased in DLBCL cells with CHST11 knockdown, suggesting that CHST11 might inhibit MOB1B expression to diminish LATS1 activation (Fig. 7G). Consistent with the preceding results [34], the total level of YAP was diminished in both the nucleus and cytoplasm upon KIAA1429 depletion, while the decreased level of YAP in the nucleus was reversed by CHST11 knockdown (Fig. 7H). Knockdown of KIAA1429 retained YAP in the cytoplasm, and further knockdown of CHST11 enhanced YAP expression in the nucleus (Fig. 7H). Furthermore, by immunofluorescence confocal imaging analysis, KIAA1429-deficient cells displayed reduced levels and nuclear localization of YAP, suggesting elevated inactivation and degradation of YAP, whereas this reduction in YAP expression induced by KIAA1429 depletion was largely abolished by CHST11 knockdown in these cells (Fig. 7I). Since dysregulation of the Hippo–YAP pathway ultimately triggers transcriptional activation of the YAP–TEAD4 complex and initiates the expression of downstream target genes, we explored whether TEAD4 transcription factor was responsible for the YAP activity regulated by KIAA1429. We found that the increase in YAP phosphorylation levels and decrease in protein levels induced by KIAA1429 knockdown were accompanied by the reduction in TEAD4 expression, whereas the increase in YAP expression induced by inhibition of CHST11 in these cells promoted TEAD4 expression (Fig. 7J). These results indicated that TEAD4 transcription factors might be a mediator of the YAP activity in DLBCL. Previous studies revealed KIAA1429 as a regulator of the TGFβ pathway. To investigate whether Smad2/3, the crucial downstream effector of TGF-β signaling, could involve in YAP transcriptional regulation, we treated DLBCL cells with ITD-1, which blocked pSmad2/3 induced by TGFβ2. Western blotting showed that the phosphorylation of Smad2 and Smad3 was inhibited upon treatment with ITD-1, whereas the expression of KIAA1429, YAP, and pYAP was not changed, indicating that Smad2/3 was not the mediator of the YAP activity regulated by KIAA1429 (Additional file 5: Fig. S4D). Taken together, our findings demonstrated that KIAA1429 knockdown contributed to the regulation of Hippo–YAP signaling by facilitating CHST11 expression in DLBCL (Additional file 6).

### KIAA1429 promotes tumorigenesis in DLBCL by inactivating YAP

**Fig. 8:**
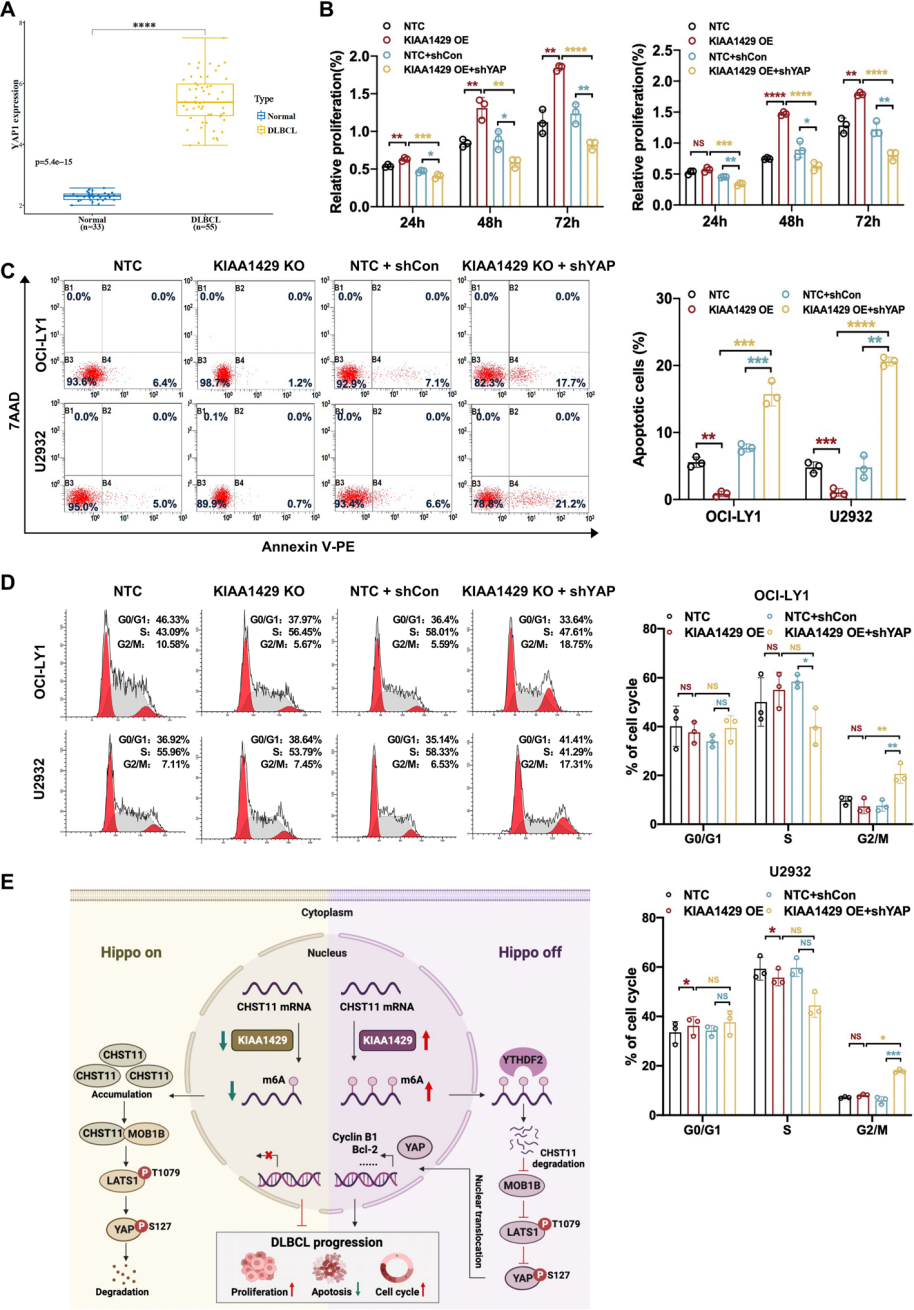
KIAA1429 inactivated Hippo–YAP signaling by interacting with CHST11. **A** *YAP* was highly expressed in DLBCL tissues (*n* = 55) compared with healthy tonsil tissues (*n* = 33) based on the GSE56315 dataset. **B**–**D** Knockdown of YAP inhibited cell proliferation promotion, apoptosis reduction, and cell cycle progression acceleration induced by KIAA1429 overexpression. Mean ± SD is presented for three independent experiments. *p < 0.05, **p < 0.01; ***p < 0.001; ****p < 0.0001. **E** Model of KIAA1429-mediated m6A modification regulating DLBCL progression through the Hippo–YAP pathway

Since KIAA1429 negatively regulated CHST11 and activation of YAP, we further explored whether this regulation was responsible for the tumor-promoting effects of KIAA1429 in DLBCL. The mRNA level of *YAP* was first determined by GSE56315 dataset, and the elevated *YAP* mRNA level was found in DLBCL sample compared with the normal B cells (Fig. 8A). Subsequently, lentivirus-mediated *YAP* shRNA to inhibit YAP expression was performed to investigate the functional significance of YAP in KIAA1429 effect, and the knockdown efficacy was validated by western blotting (Additional file 5: Fig. S4E). As expected, knockdown of YAP reversed the proliferation promotion induced by KIAA1429 overexpression (Fig. 8B). Additionally, YAP knockdown rescued the effects of KIAA1429 overexpression on apoptosis (Fig. 8C) and cell cycle progression (Fig. 8D), thereby exerting tumor-suppressive effects in DLBCL. Overall, these results indicated that KIAA1429 played a role in promoting DLCBL tumorigenesis by inhibiting YAP activation.

## Discussion

In the current study, dysregulated expression levels of m6A regulators were demonstrated and correlated with poor outcomes in DLBCL. Moreover, the m6A methyltransferase KIAA1429 was identified to be upregulated in patients with DLBCL for the first time, and its elevated expression suggested a worse clinical outcome, especially in the GCB subtype. Targeting KIAA1429 in DLBCL presented potential antitumor effects in repression of cell proliferation and induction of cell cycle arrest and apoptosis. KIAA1429 stimulated the m6A modification of *CHST11* mRNA, which then recruited YTHDF2 to bind to the m6A site and inhibited *CHST11* stability and expression to interfere with the interaction between CHST11 and MOB1B, resulting in inhibited activation of Hippo–YAP signaling, thereby reprogramming the expression of Hippo target genes that regulate cell proliferation, cell cycle, and apoptosis. Our study uncovered a novel dimension of epigenetic alteration that results in the progression of DLBCL and affects its prognosis.

As the most abundant RNA modification, m6A modification displays vital and different biological functions in multiple tumor progression [26, 35–37]. Although studies revealed that the m6A methyltransferase exhibits oncogenic activity in DLBCL [20, 38], there is little direct evidence showing other modulators as oncogenic or tumor suppressive effects. Since m6A regulators are highly heterogeneous in their genetic and expression profiles in different cancer contexts [39–41], we explored whether aberrant methylation of m6A was associated with DLBCL progression and prognosis. Our study observed significant differences in m6A regulator expression in DLBCL. A novel risk-prediction model based on the m6A regulators with prognostic value was constructed, and we found that, in the GCB subtype of DLBCL, high-risk scores were relevant to poor prognosis. Additionally, highly expressed *KIAA1429* showed worse clinical outcomes in patients with DLBCL with DHL, who exhibited a limited response to standard R-CHOP chemoimmunotherapy and tended to present poor prognosis [23], suggesting that KIAA1429 may be a predictor to guide the prognosis of DLBCL. Previous studies have demonstrated the oncogenic role of KIAA1429 in various human cancers, including gastric cancer [42], non-small cell lung cancer [43], breast cancer [44], and liver cancer [45], but not in DLBCL. Our results revealed that KIAA1429 expression was observed at high levels in DLBCL patient specimens and correlated with the clinical features and poor prognosis. Furthermore, knockout of KIAA1429 remarkably suppressed cell proliferation, induced cell cycle arrest in the G2/M phase, facilitated cell apoptosis in vitro, and repressed tumor growth in vivo, which verified the tumorigenic effects of KIAA1429 in DLBCL. Our findings not only revealed the potential value of m6A regulators in predicting the poor prognosis of patients with DLBCL but also provided evidence for KIAA1429 as a prognostic biomarker and therapeutic target in DLBCL.

KIAA1429 acts as an essential element of m6A methyltransferase complex and commonly catalyzes m6A modification near the 3′ UTR and stop codon of target mRNA [24, 46]. Indeed, higher levels of m6A were observed upon KIAA1429 overexpression in our study, highlighting the indispensable effect of KIAA1429 on m6A modification. On the basis of MeRIP-seq and RNA-seq, we found CHST11 to be a crucial downstream target of KIAA1429 in DLBCL. Suppression of KIAA1429 decreased the m6A level of *CHST11*, resulting in elevated expression of CHST11. Additionally, we identified an epigenetically mediated mechanism whereby KIAA1429 repressed CHST11 expression in an m6A-YTHDF2-dependent manner. Direct interaction between YTHDF2 and *CHST11* was observed, and the expression of *CHST11* was negatively associated with *YTHDF2*. As an enzyme regulating cartilage proteoglycan, CHST11 plays a critical role in development of mammalian chondrocytes and has not received much attention in cancer initiation and development. Limited studies have indicated that CHST11 expression is enhanced in aggressive breast cancers [47, 48], multiple myeloma [49], and ovarian cancer [50]. However, other research revealed that CHST11 expression was decreased with increasing colorectal cancer stage [51], and expression of CHST11 diminished in hepatocellular carcinoma correlated positively with malignancy [52]. Here, our study was the first to reveal the in vitro and in vivo anti-tumor roles of CHST11 in DLBCL. Moreover, we observed that CHST11 inhibition exerted opposing effects on cell proliferation suppression, cell cycle arrest, and apoptosis activation induced by KIAA1429 depletion. Our results provide a novel regulatory model of KIAA1429 in DLBCL progression.

Our previous study demonstrated the critical role of Hippo–YAP signaling in DLBCL progression [53]. Disruption of Hippo–YAP signaling has been confirmed to promote tumorigenesis in breast cancer [54, 55], ovarian cancer [56, 57], neurofibroma [58], and prostate cancer [59, 60]. In this study, we observed that KIAA1429-mediated Hippo–YAP pathway dysregulation also contributed to the initiation and progression of DLBCL. CHST11 has been reported to be positively modulated by activating the TGFβ signaling cascade in a cell type-specific manner through multiple *cis*-regulatory elements [61]. Suppression of oncogenic HRAS signaling in Costello syndrome fibroblasts increases CHST11 expression [62]. In addition, the activity of the MAPK pathway in HCC cells is enhanced upon CHST11 inhibition [52]. These studies indicated that CHST11 might exert crucial roles in cancer progression by functioning as an upstream regulator or downstream target in signaling pathways. Our study demonstrated that KIAA1429 might facilitate DLBCL progression by inhibiting CHST11 expression and attenuating the activation of Hippo–YAP signaling caused by the interaction of CHST11 with MOB1B. MOB1B is identified as a tumor suppressor, and it interacts with and activates LATS1/2 kinase, which is critical for fully activating LATS1 [33]. YAP is phosphorylated by the MOB1/LATS kinase complex resulting in its retention in the cytoplasm and subsequent degradation [34]. In our study, we found that direct interaction of CHST11 and MOB1B and KIAA1429 knockdown facilitated the activation of the Hippo pathway and cytosol retention or degradation of YAP, which was reversed by CHST11 knockdown. Further elucidation of how CHST11 regulates MOB1B expression is needed to provide insight into the unique regulatory mechanism of KIAA1429 on the Hippo–YAP pathway in DLBCL.

## Conclusions

Our study demonstrated that YTHDF2 bound KIAA1429-mediated m6A modification of *CHST11* mRNA and diminished expression of CHST11, identifying a previously unknown mechanism in which the Hippo–YAP pathway inactivation is epigenetically regulated in DLBCL. Furthermore, patients with DLBCL with elevated KIAA1429 expression presented reduced CHST11 levels and a poor prognosis. Targeting KIAA1429 to the critical regulation of *CHST11* by YTHDF2-coupled m6A modification may provide a promising therapeutic strategy for patients with DLBCL.

## Supplementary Information

**Additional file 1: Table S1. **List of sequences used in this study. **Table S2. **List of primers used in this study. **Table S3. **Results of correlation analysis between expression of m6A regulators and the clinicopathological features of DLBCL. **Table S4. **Results of KEGG enrichment analysis of genes associated with KIAA1429 upregulation.

**Additional file 2: Figure S1. **Identification of KIAA1429 as a prognostic marker for DLBCL.

**Additional file 3: Figure S2. **Weighted gene co-expression network analysis (WGCNA) in patients with DLBCL.

**Additional file 4:  Figure S3. **Identified the downstream target of KIAA1429.

**Additional file 5:  Figure S4. **The molecular mechanism of KIAA1429 regulation in DLBCL.

**Additional file 6: Figure S5. **Original images of western blotting analysis.

## Data Availability

The datasets used and/or analyzed in our study are available from the GEO database (https://www.ncbi.nlm.nih.gov/geo/) and the TCGA database (https://portal.gdc.cancer.gov/). The original images of western blotting analysis in this study are included in Additional file 6. The additional data supporting the findings of this study could be obtained from the corresponding author upon reasonable request.

## Abbreviations

CHST11: Carbohydrate sulfotransferase 11
CDSs: Coding sequences
Co-IP: Coimmunoprecipitation
DEGs: Differentially expressed genes
DHL: Double-hit lymphoma
DLBCL: Diffuse large B-cell lymphoma
DIRAS1: DIRAS family GTPase 1
GEO: Gene Expression Omnibus
GO: Gene Ontology
H&E: Hematoxylin and eosin
IHC: Immunohistochemical
IPI: International Prognostic Index
KEGG: Kyoto Encyclopedia of Genes and Genomes
KO: Knockout
m6A: *N*^6^-methyladenosine
MeRIP-seq: Methylated RNA immunoprecipitation sequencing
OS: Overall survival
RHL: Reactive hyperplasias of lymph nodes
RIP: RNA immunoprecipitation
RNA-seq: RNA sequencing
RT-qPCR: Quantitative real-time polymerase chain reaction
TCGA: The Cancer Genome Atlas
USP31: Ubiquitin-specific peptidase 31
UTRs: Untranslated regions
VIRMA: Alias KIAA1429, virus-like m6A methyltransferase-associated protein
WGCNA: Weighted gene co-expression network analysis
YAP: Alias YAP1, Yes-Associated Protein
YTHDF: YTH domain family

## References

[1] Shi Q, Schmitz N, Ou FS, Dixon JG, Cunningham D, Pfreundschuh M (2018). Progression-free survival as a surrogate end point for overall survival in first-line diffuse large B-cell lymphoma: an individual patient-level analysis of multiple randomized trials (SEAL). J Clin Oncol.

[2] Sehn LH, Salles G (2021). Diffuse large B-cell lymphoma. N Engl J Med.

[3] Hu S, Ren S, Cai Y, Liu J, Han Y, Zhao Y (2021). Glycoprotein PTGDS promotes tumorigenesis of diffuse large B-cell lymphoma by MYH9-mediated regulation of Wnt-beta-catenin-STAT3 signaling. Cell Death Differ.

[4] Sun JR, Zhang X, Zhang Y (2019). MiR-214 prevents the progression of diffuse large B-cell lymphoma by targeting PD-L1. Cell Mol Biol Lett.

[5] Ramos JC, Sparano JA, Chadburn A, Reid EG, Ambinder RF, Siegel ER (2020). Impact of Myc in HIV-associated non-Hodgkin lymphomas treated with EPOCH and outcomes with vorinostat (AMC-075 trial). Blood.

[6] Velcheti V, Wong KK, Saunthararajah Y (2019). EZH2 inhibitors: take it EZy, it is all about context. Cancer Discov.

[7] Kuhnl A, Cunningham D, Chau I (2017). Beyond genomics—targeting the epigenome in diffuse large B-cell lymphoma. Cancer Treat Rev.

[8] Cai Y, Feng R, Lu T, Chen X, Zhou X, Wang X (2021). Novel insights into the m(6)A-RNA methyltransferase METTL3 in cancer. Biomark Res..

[9] Chen X, Zhou X, Wang X (2022). m(6)A binding protein YTHDF2 in cancer. Exp Hematol Oncol.

[10] Sun W, Li Y, Ma D, Liu Y, Xu Q, Cheng D (2022). ALKBH5 promotes lung fibroblast activation and silica-induced pulmonary fibrosis through miR-320a-3p and FOXM1. Cell Mol Biol Lett.

[11] Knuckles P, Lence T, Haussmann IU, Jacob D, Kreim N, Carl SH (2018). Zc3h13/Flacc is required for adenosine methylation by bridging the mRNA-binding factor Rbm15/Spenito to the m(6)A machinery component Wtap/Fl(2)d. Genes Dev.

[12] Yue Y, Liu J, Cui X, Cao J, Luo G, Zhang Z (2018). VIRMA mediates preferential m(6)A mRNA methylation in 3′UTR and near stop codon and associates with alternative polyadenylation. Cell Discov..

[13] Zeng J, Zhang H, Tan Y, Wang Z, Li Y, Yang X (2021). Genetic alterations and functional networks of m6A RNA methylation regulators in pancreatic cancer based on data mining. J Transl Med.

[14] Chen M, Wei L, Law CT, Tsang FH, Shen J, Cheng CL (2018). RNA N6-methyladenosine methyltransferase-like 3 promotes liver cancer progression through YTHDF2-dependent posttranscriptional silencing of SOCS2. Hepatology.

[15] Dai YZ, Liu YD, Li J, Chen MT, Huang M, Wang F (2022). METTL16 promotes hepatocellular carcinoma progression through downregulating RAB11B-AS1 in an m(6)A-dependent manner. Cell Mol Biol Lett.

[16] Ni Z, Sun P, Zheng J, Wu M, Yang C, Cheng M (2022). JNK signaling promotes bladder cancer immune escape by regulating METTL3-mediated m6A modification of PD-L1 mRNA. Cancer Res.

[17] Wang Q, Chen C, Ding Q, Zhao Y, Wang Z, Chen J (2020). METTL3-mediated m(6)A modification of HDGF mRNA promotes gastric cancer progression and has prognostic significance. Gut.

[18] Wang X, Wang J, Tsui YM, Shi C, Wang Y, Zhang X (2021). RALYL increases hepatocellular carcinoma stemness by sustaining the mRNA stability of TGF-beta2. Nat Commun.

[19] Miao R, Dai CC, Mei L, Xu J, Sun SW, Xing YL (2020). KIAA1429 regulates cell proliferation by targeting c-Jun messenger RNA directly in gastric cancer. J Cell Physiol.

[20] Han H, Fan G, Song S, Jiang Y, Qian C, Zhang W (2021). piRNA-30473 contributes to tumorigenesis and poor prognosis by regulating m6A RNA methylation in DLBCL. Blood.

[21] Xie Z, Li M, Hong H, Xu Q, He Z, Peng Z (2021). Expression of N(6)-methyladenosine (m(6)A) regulators correlates with immune microenvironment characteristics and predicts prognosis in diffuse large cell lymphoma (DLBCL). Bioengineered.

[22] Sha C, Barrans S, Cucco F, Bentley MA, Care MA, Cummin T (2019). Molecular high-grade B-cell lymphoma: defining a poor-risk group that requires different approaches to therapy. J Clin Oncol.

[23] Landsburg DJ, Falkiewicz MK, Maly J, Blum KA, Howlett C, Feldman T (2017). Outcomes of patients with double-hit lymphoma who achieve first complete remission. J Clin Oncol.

[24] Wen J, Lv R, Ma H, Shen H, He C, Wang J (2018). Zc3h13 regulates nuclear RNA m(6)A methylation and mouse embryonic stem cell self-renewal. Mol Cell.

[25] Wang W, Shao F, Yang X, Wang J, Zhu R, Yang Y (2021). METTL3 promotes tumour development by decreasing APC expression mediated by APC mRNA N(6)-methyladenosine-dependent YTHDF binding. Nat Commun.

[26] Zhang Z, Luo K, Zou Z, Qiu M, Tian J, Sieh L (2020). Genetic analyses support the contribution of mRNA N(6)-methyladenosine (m(6)A) modification to human disease heritability. Nat Genet.

[27] Hou Y, Zhang Q, Pang W, Hou L, Liang Y, Han X (2021). YTHDC1-mediated augmentation of miR-30d in repressing pancreatic tumorigenesis via attenuation of RUNX1-induced transcriptional activation of Warburg effect. Cell Death Differ.

[28] Cai Z, Zhang Y, Yang L, Ma C, Fei Y, Ding J (2022). ALKBH5 in mouse testicular Sertoli cells regulates Cdh2 mRNA translation to maintain blood-testis barrier integrity. Cell Mol Biol Lett.

[29] Lee SH, Singh I, Tisdale S, Abdel-Wahab O, Leslie CS, Mayr C (2018). Widespread intronic polyadenylation inactivates tumour suppressor genes in leukaemia. Nature.

[30] Schmidt HH, Dyomin VG, Palanisamy N, Itoyama T, Nanjangud G, Pirc-Danoewinata H (2004). Deregulation of the carbohydrate (chondroitin 4) sulfotransferase 11 (CHST11) gene in a B-cell chronic lymphocytic leukemia with a t(12;14)(q23;q32). Oncogene.

[31] Lee YA, Noon LA, Akat KM, Ybanez MD, Lee TF, Berres ML (2018). Autophagy is a gatekeeper of hepatic differentiation and carcinogenesis by controlling the degradation of Yap. Nat Commun.

[32] Watt KI, Henstridge DC, Ziemann M, Sim CB, Montgomery MK, Samocha-Bonet D (2021). Yap regulates skeletal muscle fatty acid oxidation and adiposity in metabolic disease. Nat Commun.

[33] Jin J, Zhang L, Li X, Xu W, Yang S, Song J (2022). Oxidative stress-CBP axis modulates MOB1 acetylation and activates the Hippo signaling pathway. Nucleic Acids Res.

[34] Song J, Wang T, Chi X, Wei X, Xu S, Yu M (2019). Kindlin-2 inhibits the hippo signaling pathway by promoting degradation of MOB1. Cell Rep.

[35] Fang R, Chen X, Zhang S, Shi H, Ye Y, Shi H (2021). EGFR/SRC/ERK-stabilized YTHDF2 promotes cholesterol dysregulation and invasive growth of glioblastoma. Nat Commun.

[36] Yin H, Zhang X, Yang P, Zhang X, Peng Y, Li D (2021). RNA m6A methylation orchestrates cancer growth and metastasis via macrophage reprogramming. Nat Commun.

[37] Wang L, Yi X, Xiao X, Zheng Q, Ma L, Li B (2022). Exosomal miR-628-5p from M1 polarized macrophages hinders m6A modification of circFUT8 to suppress hepatocellular carcinoma progression. Cell Mol Biol Lett.

[38] Cheng Y, Fu Y, Wang Y, Wang J (2020). The m6A methyltransferase METTL3 is functionally implicated in DLBCL development by regulating m6A modification in PEDF. Front Genet.

[39] Tang Y, Chen K, Song B, Ma J, Wu X, Xu Q (2021). m6A-Atlas: a comprehensive knowledgebase for unraveling the N6-methyladenosine (m6A) epitranscriptome. Nucleic Acids Res.

[40] Li ZX, Zheng ZQ, Yang PY, Lin L, Zhou GQ, Lv JW (2022). WTAP-mediated m(6)A modification of lncRNA DIAPH1-AS1 enhances its stability to facilitate nasopharyngeal carcinoma growth and metastasis. Cell Death Differ.

[41] Ding H, Zhang X, Su Y, Jia C, Dai C (2020). GNAS promotes inflammation-related hepatocellular carcinoma progression by promoting STAT3 activation. Cell Mol Biol Lett.

[42] Zhu Z, Zhou Y, Chen Y, Zhou Z, Liu W, Zheng L (2022). m(6)A methyltransferase KIAA1429 regulates the cisplatin sensitivity of gastric cancer cells via stabilizing FOXM1 mRNA. Cancers (Basel)..

[43] Zhou B, Bie F, Zang R, Zhang M, Song P, Liu L (2022). RNA modification writer expression profiles predict clinical outcomes and guide neoadjuvant immunotherapy in non-small cell lung cancer. EBioMedicine.

[44] Qian JY, Gao J, Sun X, Cao MD, Shi L, Xia TS (2019). KIAA1429 acts as an oncogenic factor in breast cancer by regulating CDK1 in an N6-methyladenosine-independent manner. Oncogene.

[45] Lan T, Li H, Zhang D, Xu L, Liu H, Hao X (2019). KIAA1429 contributes to liver cancer progression through N6-methyladenosine-dependent post-transcriptional modification of GATA3. Mol Cancer.

[46] Zhang Q, Kang Y, Wang S, Gonzalez GM, Li W, Hui H (2021). HIV reprograms host m(6)Am RNA methylome by viral Vpr protein-mediated degradation of PCIF1. Nat Commun.

[47] Cooney CA, Jousheghany F, Yao-Borengasser A, Phanavanh B, Gomes T, Kieber-Emmons AM (2011). Chondroitin sulfates play a major role in breast cancer metastasis: a role for CSPG4 and CHST11 gene expression in forming surface P-selectin ligands in aggressive breast cancer cells. Breast Cancer Res.

[48] Herman D, Leakey TI, Behrens A, Yao-Borengasser A, Cooney CA, Jousheghany F (2015). CHST11 gene expression and DNA methylation in breast cancer. Int J Oncol.

[49] Bret C, Hose D, Reme T, Sprynski AC, Mahtouk K, Schved JF (2009). Expression of genes encoding for proteins involved in heparan sulphate and chondroitin sulphate chain synthesis and modification in normal and malignant plasma cells. Br J Haematol.

[50] ten Dam GB, van de Westerlo EM, Purushothaman A, Stan RV, Bulten J, Sweep FC (2007). Antibody GD3G7 selected against embryonic glycosaminoglycans defines chondroitin sulfate-E domains highly up-regulated in ovarian cancer and involved in vascular endothelial growth factor binding. Am J Pathol.

[51] Kalathas D, Theocharis DA, Bounias D, Kyriakopoulou D, Papageorgakopoulou N, Stavropoulos MS (2009). Alterations of glycosaminoglycan disaccharide content and composition in colorectal cancer: structural and expressional studies. Oncol Rep.

[52] Zhou H, Li Y, Song X, Zhao Y, Cheng L, Zhao L (2016). CHST11/13 regulate the metastasis and chemosensitivity of human hepatocellular carcinoma cells via mitogen-activated protein kinase pathway. Dig Dis Sci.

[53] Zhou X, Chen N, Xu H, Zhou X, Wang J, Fang X (2020). Regulation of Hippo-YAP signaling by insulin-like growth factor-1 receptor in the tumorigenesis of diffuse large B-cell lymphoma. J Hematol Oncol.

[54] Ma S, Wu Z, Yang F, Zhang J, Johnson RL, Rosenfeld MG (2021). Hippo signalling maintains ER expression and ER(+) breast cancer growth. Nature.

[55] Zhang Z, Du J, Wang S, Shao L, Jin K, Li F (2019). OTUB2 promotes cancer metastasis via Hippo-independent activation of YAP and TAZ. Mol Cell.

[56] Hua G, Lv X, He C, Remmenga SW, Rodabough KJ, Dong J (2016). YAP induces high-grade serous carcinoma in fallopian tube secretory epithelial cells. Oncogene.

[57] He C, Lv X, Huang C, Hua G, Ma B, Chen X (2019). YAP1-LATS2 feedback loop dictates senescent or malignant cell fate to maintain tissue homeostasis. EMBO Rep.

[58] Chen Z, Mo J, Brosseau JP, Shipman T, Wang Y, Liao CP (2019). Spatiotemporal loss of NF1 in Schwann cell lineage leads to different types of cutaneous neurofibroma susceptible to modification by the Hippo pathway. Cancer Discov.

[59] Han Y, Zhang L, Yu X, Wang S, Xu C, Yin H (2021). Retraction note: alginate oligosaccharide attenuates alpha2,6-sialylation modification to inhibit prostate cancer cell growth via the Hippo/YAP pathway. Cell Death Dis.

[60] Wang G, Lu X, Dey P, Deng P, Wu CC, Jiang S (2016). Targeting YAP-dependent MDSC infiltration impairs tumor progression. Cancer Discov.

[61] Gronau T, Kruger K, Prein C, Aszodi A, Gronau I, Iozzo RV (2017). Forced exercise-induced osteoarthritis is attenuated in mice lacking the small leucine-rich proteoglycan decorin. Ann Rheum Dis.

[62] Kluppel M, Samavarchi-Tehrani P, Liu K, Wrana JL, Hinek A (2012). C4ST-1/CHST11-controlled chondroitin sulfation interferes with oncogenic HRAS signaling in Costello syndrome. Eur J Hum Genet.

