# Supplementary material for: KIAA1429-mediated m6A modification of CHST11 promotes progression of diffuse large B-cell lymphoma by regulating Hippo–YAP pathway
Source: Cell Mol Biol Lett. 2023 Apr 19;28:32. doi: 10.1186/s11658-023-00445-w (PMC10114474; doi:10.1186/s11658-023-00445-w)
Supplement: Supplementary file 1 — Additional file 1: Table S1. List of sequences used in this study. Table S2. List of primers used in this study. Table S3. Results of correlation analysis between expression of m6A regulators and the clinicopathological features of DLBCL. Table S4. Results of KEGG enrichment analysis of genes associated with KIAA1429 upregulation. [file 11658_2023_445_MOESM1_ESM.docx]

**Figure S1.** Identification of KIAA1429 as a prognostic marker for DLBCL.

**Figure S2.** Weighted gene co-expression network analysis (WGCNA) in DLBCL patients.

**Figure S3.** Identified the downstream target of KIAA1429.

**Figure S4.** The molecular mechanism of KIAA1429 regulation in DLBCL.

**Figure S5.** Original images of western blotting analysis.

**Table S1.** List of sequences used in this study.

**Table S2.** List of primers used in this study.

**Table S3.** Results of correlation analysis between expression of m6A regulators and the clinicopathological features of DLBCL.

**Table S4.** Results of KEGG enrichment analysis of genes associated with KIAA1429 upregulation.

**Figure S1. Identification of KIAA1429 as a prognostic marker for DLBCL. A** Correlation analysis among m6A regulators based on GSE117556 dataset. **B** The univariate Cox regression analysis showed the genes significantly associated with OS. **C** the LASSO coefficient profiles. Each curve corresponds to a certain gene. It shows the path of its coefficient against the L1-norm of the whole coefficient vector at various λ values of each gene. **D** illustrates partial likelihood deviance for the LASSO coefficient profiles. The red dotted line stands for the cross-validation curve, error bars represent the upper and lower standard deviation curves along the λ sequence. The left vertical line shows the optimal λ value at which the minimum mean squared error is achieved and the corresponding genes. The right vertical line is for the most regularized model whose mean squared error is within 1 standard error of the minimal. It is indicated that the genes identified by optimal λ (log λ = −4) are the simplest model with the best performance. The axis above in supplementary Figures S1C and D indicates the number of genes involved in the LASSO model. **C-D** LASSO Cox regression using 19 m6A regulators to obtain 6 genes based on the minimum criteria to construct the risk model from the GEO database (GSE117556). **E** Kaplan-Meier curves revealed better survival in the low-risk group, and a log-rank test was used to compare the median overall survival of DLBCL patients in high-risk and low-risk groups. **F** AUC values of ROC curves to assess the predictive capability of risk scores. **G**-**H** The univariate and multivariate analyses exhibited that risk score was an independent prognostic marker for OS in DLBCL. **I** Higher KIAA1429 expression was found in DHL patients than in non-DHL patients. **J** Elevated levels of KIAA1429 were correlated with a higher IPI score. **K** Kaplan-Meier survival curves showed no statistical difference in the relevance between KIAA1429 expression and survival time of non-DHL patients. **L** Univariate Cox regression analysis to assess the association between KIAA1429 expression and OS.

**Figure S2. Weighted gene co-expression network analysis (WGCNA) in DLBCL patients. A** The heatmap of the TOM among 1000 selected genes in WGCNA. **B** The heatmap showed the top 40 genes associated with KIAA1429

**Figure S3. Identified the downstream target of KIAA1429. A** Distribution of differentially expressed genes from RNA-seq. **B** Volcano plots displaying distribution of transcripts with significantly altered m6A peaks in KIAA1429 depletion cells relative to control OCI-LY1 cells. **C** Venn diagram was used to examine overlaps of differentially expressed genes between RNA-seq and MeRIP-seq. **D-E** The mRNA levels of USP31 and DIRAS1 in DLBCL cells compared to normal B cells. Data from three independent experiments are presented as the mean ± SD. *p < 0.05

**Figure S4. The molecular mechanism of KIAA1429 regulation in DLBCL. A-B** GO and KEGG analyses of differentially expressed genes in MeRIP-seq. **C** Visualization of the correlation between CHST11 and Hippo-YAP pathway core components. **D** Western blotting exhibited that after treatment with ITD-1, the protein levels of P-Smad2 and P-Smad3 in DLBCL cells were reduced, while the protein expression of YAP and KIAA1429 were not altered. **E** Efficiency validation of KIAA1429 overexpression and YAP knockdown by western blotting.

| **Table S1. List of sequences used in this study.** | |
| --- | --- |
| **Names** | **Sequences** |
| shKIAA1429#1 | 5′-cgGAATATGAAGCAACAAATT-3′ |
| shKIAA1429#2 | 5′-cgCTGAGCAAAGTTCTCATAT-3′ |
| shKIAA1429#3 | 5′-TTGCTGATCACGTATCATCTT-3′ |
| KIAA1429 KO#1 | 5′-GGACAACTGAAGTATAAGAG-3′ |
| KIAA1429 KO#2 | 5′-GAACATTTAAGGCGATAGGT-3′ |
| KIAA1429 KO#3 | 5′-GCATGGTCCAGTCTACCAAC-3′ |
| shCHST11#1 | 5′-GACCCTGAACCAGTACAGCAT-3′ |
| shCHST11#2 | 5′-GGCCACTTGCTTGGGATCCTT-3′ |
| shCHST11#3 | 5′-CGGGAGGAGCCTTTCAACGAA-3′ |
| shYTHDF2 | 5′-TTCCTACCAGATGCAATGTTT-3′ |
| shYAP | 5'-CCCAGTTAAATGTTCACCAAT-3' |

| **Table S2. List of primers used in this study.** | |
| --- | --- |
| **Primer names** | **Sequences** |
| HNRNPC forward | 5’-GCAACGTTACCAACAAGACAGA-3’ |
| HNRNPC reverse | 5’-TTATGAACAGAGCAGCCCACA-3’ |
| HNRNPA2B1 forward | 5’-AGACTGTGTGGTAATGAGGGA-3’ |
| HNRNPA2B1 reverse | 5’-GCTACAGCACGTTTTGGCTC-3’ |
| HNRNPG forward | 5’-CATTGGTGGGCTTAATACGGA-3’ |
| HNRNPG reverse | 5’-GCTGCATCCTTAGCGTCTG-3’ |
| YTHDC1 forward | 5’-AGGAAAGTCAGCCACAGAGT-3’ |
| YTHDC1 reverse | 5’-GCGTAGGAGATTTGGCCCTC-3’ |
| YTHDC2 forward | 5’-GGCCTCTCAAAAACGTGCAG-3’ |
| YTHDC2 reverse | 5’-CTCCCACCCATCACTTCGTG-3’ |
| YTHDF3 forward | 5’-GCTGGGTAGCTCCTCGTAAC-3’ |
| YTHDF3 reverse | 5’-AGCACGGGATGCACTTCTAC-3’ |
| IGF2BP1 forward | 5’-GCTCCTTTATGCAGGCTCCC-3’ |
| IGF2BP1 reverse | 5’-TTGGAGTCAGGTGTTTCGGG-3’ |
| IGF2BP2 forward | 5’-CACACAATGGCTTGGTTGGAA-3’ |
| IGF2BP2 reverse | 5’-AGTGATGGTTCTTTCCGGGTT-3’ |
| IGF2BP3 forward | 5’-TCCCAGCTCTATCAGTCGGT-3’ |
| IGF2BP3 reverse | 5’-CTTCCCTGAGCCTTGAACTG-3’ |
| GAPDH forward | 5’-GCACCGTCAAGGCTGAGAAC-3’ |
| GAPDH reverse | 5’-TGGTGAAGACGCCAGTGGA-3’ |
| YTHDF2 forward | 5’-CACAGGCAAGGCCCAATAAT-3’ |
| YTHDF2 reverse | 5’-ATCTGGTAGGAAGTGGGGCT-3’ |
| YTHDF1 forward | 5’-CTTCAGCGTCAATGGGAGTG-3’ |
| YTHDF1 reverse | 5’-CCGGAGCTGGTTATTGGGTA-3’ |
| KIAA1429 forward | 5’-AGTGCCCCTGTTTTCGATAGG-3’ |
| KIAA1429 reverse | 5’-TACCAGCCTCTTAGCACCAG-3’ |
| CHST11 forward | 5’-CCAAAGTATGTTGCACCCAGTC-3’ |
| CHST11 reverse | 5’-GGACAGCAGTGTTTGAGAGC-3’ |
| USP31 forward | 5’-TGGATCAGAGCGACTCCGTA-3’ |
| USP31 reverse | 5’-GGTTTCTTTGCGAGTGAGCC-3’ |
| DIRAS1 forward | 5’-GCAACAAGTGCGATGAGACG-3’ |
| DIRAS1 reverse | 5’-GGCCGAGGTCTCCATGAAAG-3’ |

**Table S3. Results of correlation analysis between expression of m6A regulators and the clinicopathological features of DLBCL.**

| **id** | **Gender** | **Stage** | **ECOG** | **IPI** | **Hit** |
| --- | --- | --- | --- | --- | --- |
| KIAA1429 | 0.666848 | 0.600701 | 0.466156 | 0.043437 | 0.000856 |
| ALKBH5 | 0.847790 | 0.352129 | 0.009397 | 0.145433 | 0.036482 |
| HNRNPC | 0.270856 | 0.020950 | 0.926548 | 0.118984 | 0.036486 |
| METTL14 | 0.008022 | 0.467936 | 0.885601 | 0.208923 | 0.205758 |
| METTL3 | 0.912360 | 0.056861 | 0.676070 | 0.045548 | 0.938999 |
| WTAP | 0.025582 | 0.172577 | 0.259986 | 0.741580 | 0.376008 |
| YTHDC2 | 0.036522 | 0.199417 | 0.357608 | 0.399723 | 0.059943 |
| YTHDF2 | 0.076978 | 0.355248 | 0.741303 | 0.043652 | 0.086391 |
| YTHDF3 | 0.991940 | 0.473612 | 0.656351 | 0.025364 | 0.278160 |
| FTO | 0.133221 | 0.730469 | 0.832732 | 0.277756 | 0.082311 |
| HNRNPA2B1 | 0.849214 | 0.872691 | 0.267195 | 0.400774 | 0.197879 |
| IGF2BP1 | 0.581759 | 0.311706 | 0.605996 | 0.789144 | 0.504096 |
| IGF2BP2 | 0.498521 | 0.531279 | 0.498482 | 0.377204 | 0.513827 |
| IGF2BP3 | 0.155661 | 0.666074 | 0.393216 | 0.614101 | 0.685654 |
| RBM15 | 0.373990 | 0.532113 | 0.600372 | 0.463834 | 0.313897 |
| RBM15B | 0.527958 | 0.075188 | 0.390400 | 0.469861 | 0.956408 |
| YTHDC1 | 0.609568 | 0.722984 | 0.091351 | 0.584966 | 0.622416 |
| YTHDF1 | 0.452209 | 0.316774 | 0.484409 | 0.360279 | 0.104326 |
| ZC3H13 | 0.476767 | 0.299150 | 0.171263 | 0.546091 | 0.155092 |

The values in the table represent the p values of the correlation.

**Table S4. The result of KEGG enrichment analysis of genes associated with KIAA1429 upregulation.**

| **ID** | **Description** | **GeneRatio** | **BgRatio** | **pvalue** | **geneID** |
| --- | --- | --- | --- | --- | --- |
| hsa00030 | Pentose phosphate pathway | 8/561 | 30/8075 | 0.00076903 | PGM2/PGM1/RPIA/DERA/PFKM/TALDO1/ALDOB/FBP2 |
| hsa00280 | Valine, leucine and isoleucine degradation | 9/561 | 48/8075 | 0.00514928 | PCCB/MCEE/HADH/ACAT1/OXCT1/HIBCH/AUH/ALDH2/OXCT2 |
| hsa00480 | Glutathione metabolism | 10/561 | 57/8075 | 0.00535227 | GPX8/ANPEP/GPX7/RRM1/NAT8B/PRDX6/TXNDC12/GSTM3/NAT8/GSTM2 |
| hsa03030 | DNA replication | 7/561 | 36/8075 | 0.01071962 | RNASEH1/RPA3/RPA1/POLE3/MCM2/LIG1/MCM5 |
| hsa03022 | Basal transcription factors | 8/561 | 45/8075 | 0.01129443 | MNAT1/TAF13/TAF5/CCNH/ERCC3/GTF2F2/TAF4/TAF6L |
| hsa04110 | Cell cycle | 16/561 | 124/8075 | 0.01164683 | ANAPC5/TFDP1/CCNH/ANAPC4/CDKN1A/BUB1/CDK6/CDC7/ANAPC13/PTTG2/MCM2/MAD1L1/E2F1/RB1/MCM5/PKMYT1 |
| hsa05168 | Herpes simplex virus 1 infection | 48/561 | 498/8075 | 0.01192307 | ZNF250/HCFC2/ZNF611/ZNF343/ZNF823/PML/ZNF700/ZNF566/ZNF684/ZNF337/EIF2AK3/ZNF136/ZNF416/ZNF674/ZNF426/ZNF559/ZNF614/ZNF43/ZNF573/ZNF425/ZNF223/ZFP30/ZNF79/ZNF7/ZNF621/ZNF761/IFIH1/ZNF589/MAP3K7/ZNF302/ZNF141/ZNF543/ZNF26/SRC/IFNGR1/ZNF211/PPP1CC/ZNF420/ZNF584/ZNF177/ZNF680/ZNF415/ZNF25/ZNF169/ZNF554/ZNF439/ZNF529/IL1B |
| hsa03050 | Proteasome | 8/561 | 46/8075 | 0.01286627 | PSMD3/PSMC2/PSMD12/PSMB5/PSME4/PSMA2/PSMD9/PSME3 |
| hsa04726 | Serotonergic synapse | 15/561 | 115/8075 | 0.01303718 | RAF1/BRAF/CACNA1C/HTR2A/GNG12/CYP2C19/PLA2G4A/CYP2D6/GNAI3/PLA2G4C/GABRB1/KCNJ3/PLA2G4F/GNG8/ALOX12B |
