# Supplementary figures and images for: KIAA1429-mediated m6A modification of CHST11 promotes progression of diffuse large B-cell lymphoma by regulating Hippo–YAP pathway
Source: Cell Mol Biol Lett. 2023 Apr 19;28:32. doi: 10.1186/s11658-023-00445-w (PMC10114474; doi:10.1186/s11658-023-00445-w)

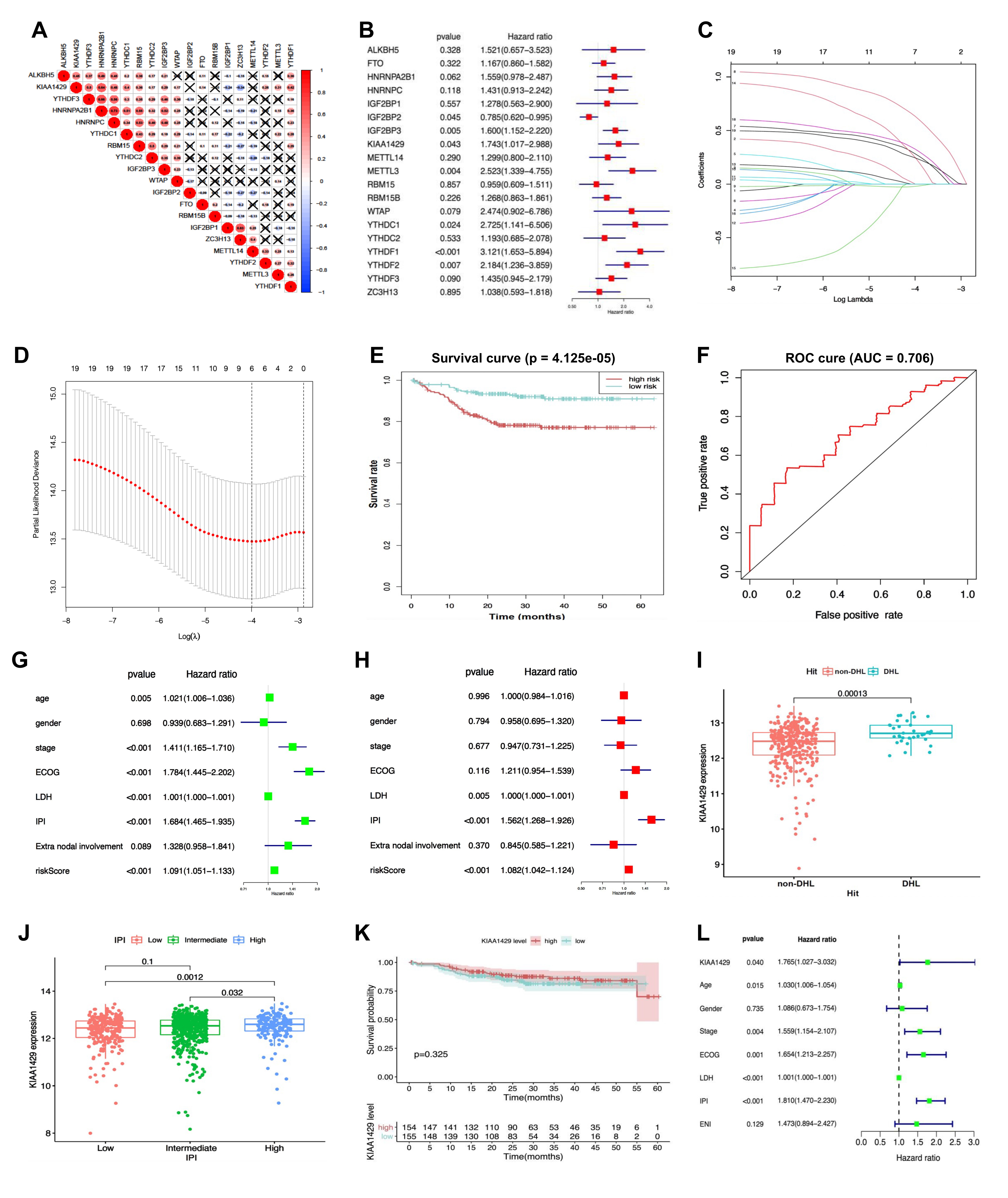

Supplement: Supplementary file 2 — Additional file 2: Figure S1. Identification of KIAA1429 as a prognostic marker for DLBCL. [file 11658_2023_445_MOESM2_ESM.jpg]

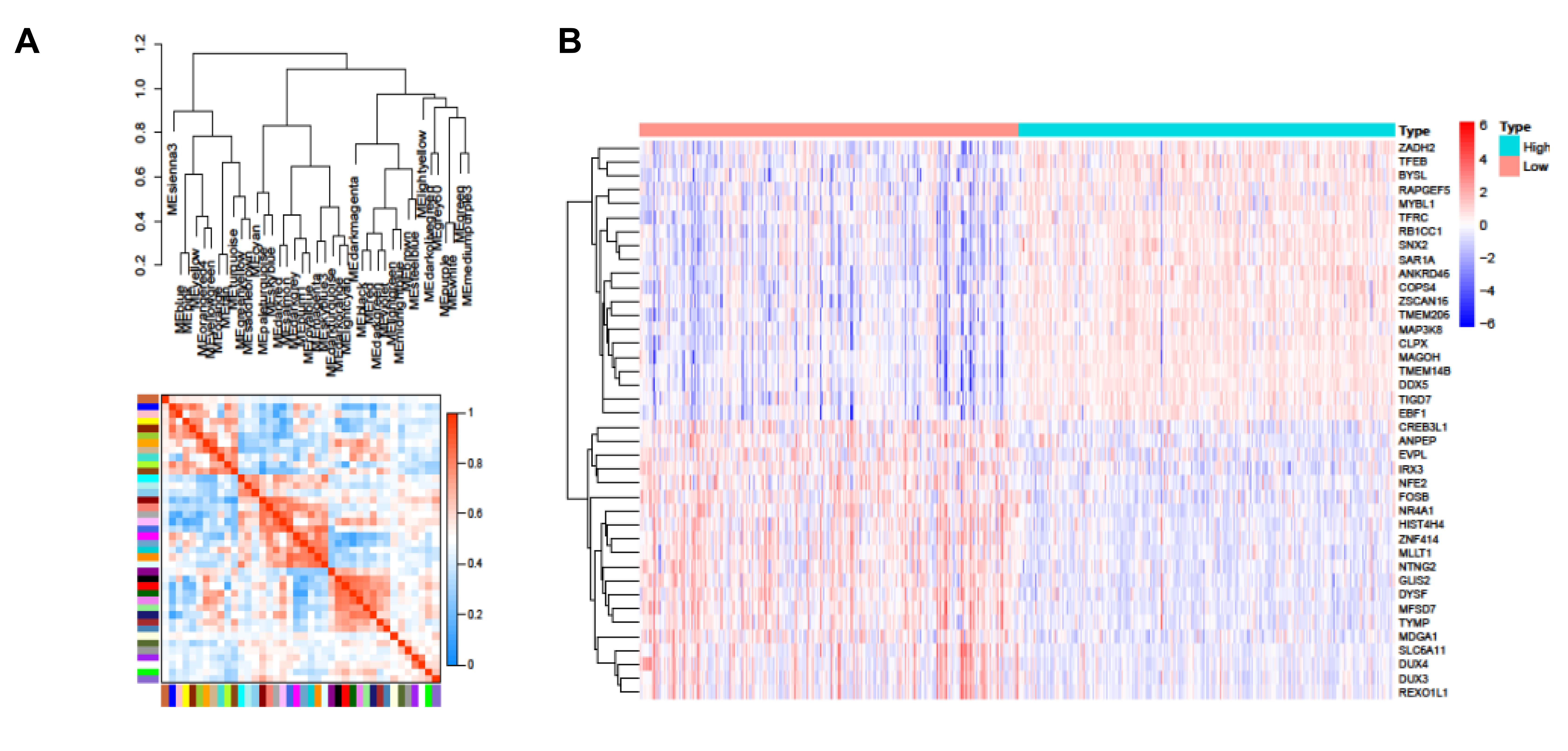

Supplement: Supplementary file 3 — Additional file 3: Figure S2. Weighted gene co-expression network analysis (WGCNA) in patients with DLBCL. [file 11658_2023_445_MOESM3_ESM.jpg]

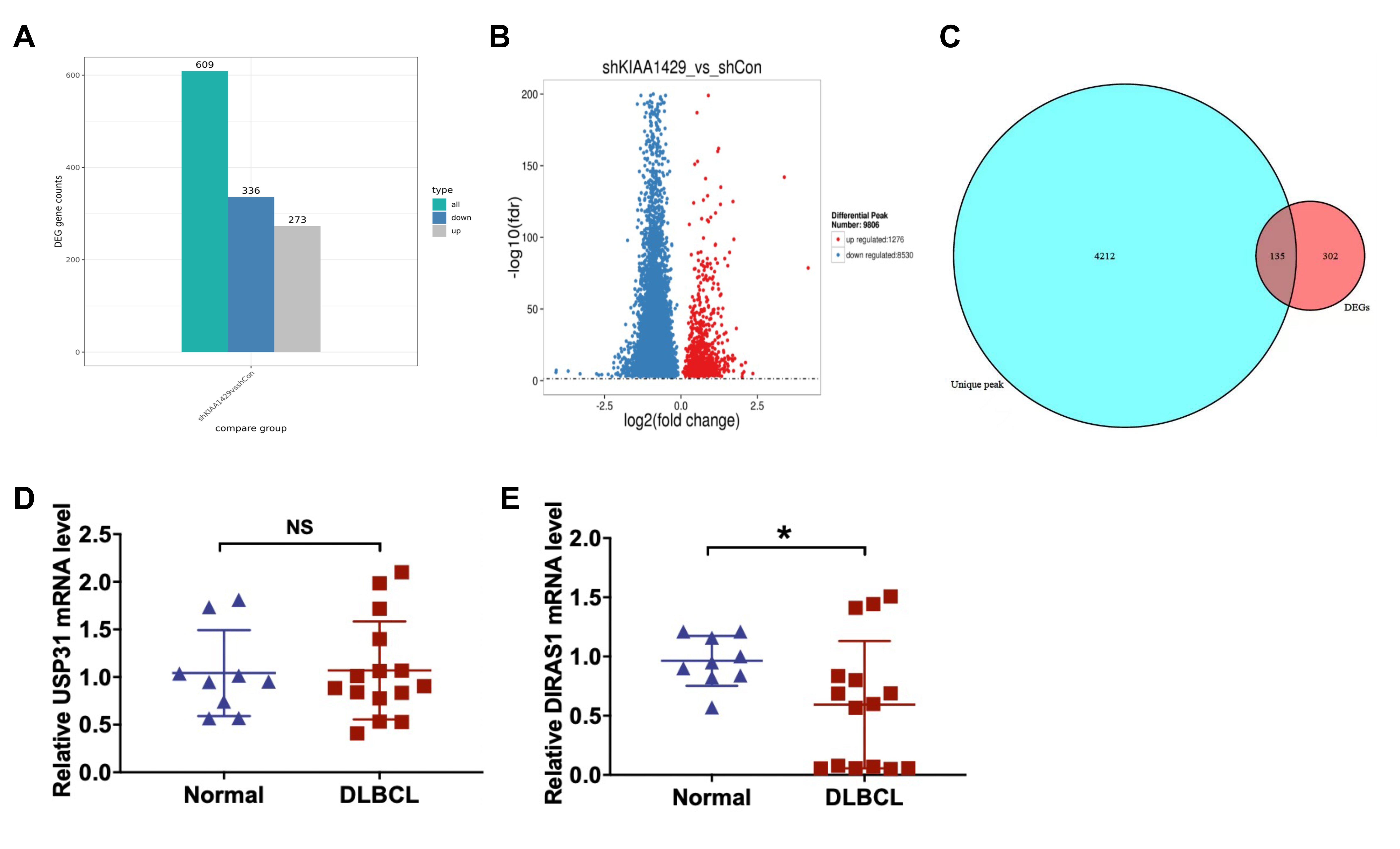

Supplement: Supplementary file 4 — Additional file 4: Figure S3. Identified the downstream target of KIAA1429. [file 11658_2023_445_MOESM4_ESM.jpg]

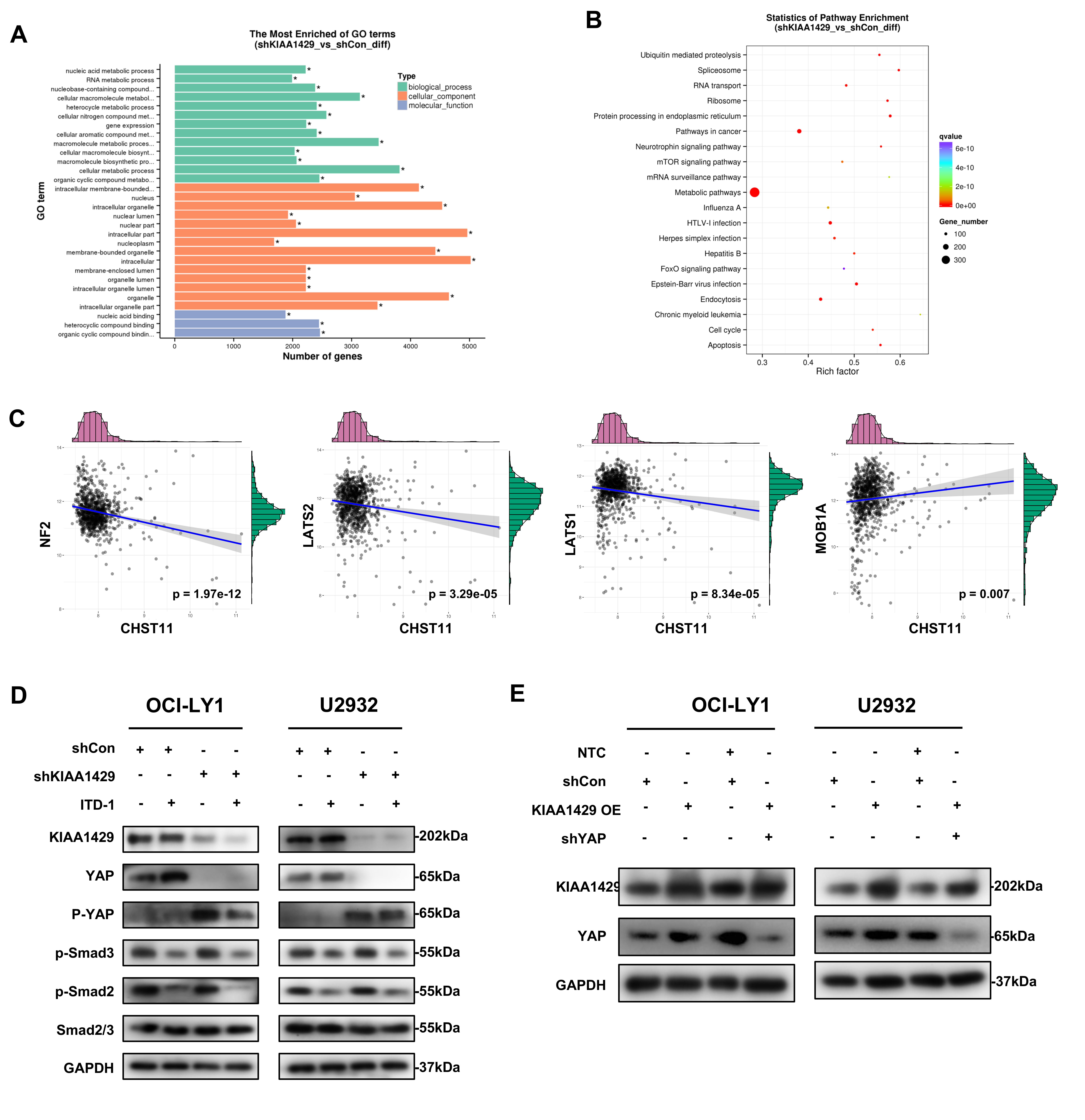

Supplement: Supplementary file 5 — Additional file 5: Figure S4. The molecular mechanism of KIAA1429 regulation in DLBCL. [file 11658_2023_445_MOESM5_ESM.jpg]
