# Supplementary material for: KIAA1429-mediated m6A modification of CHST11 promotes progression of diffuse large B-cell lymphoma by regulating Hippo–YAP pathway
Source: Cell Mol Biol Lett. 2023 Apr 19;28:32. doi: 10.1186/s11658-023-00445-w (PMC10114474; doi:10.1186/s11658-023-00445-w)
Supplement: Supplementary file 6 — Additional file 6: Figure S5. Original images of western blotting analysis. [file 11658_2023_445_MOESM6_ESM.pdf]

Figure 1D

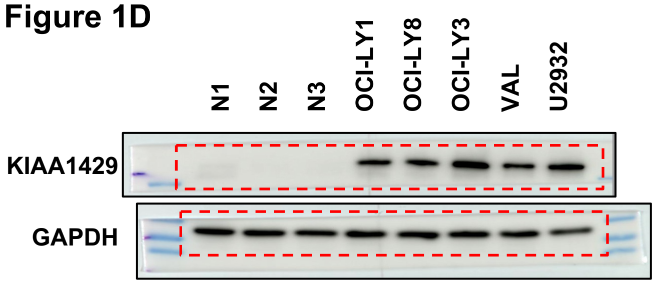

Figure 2E

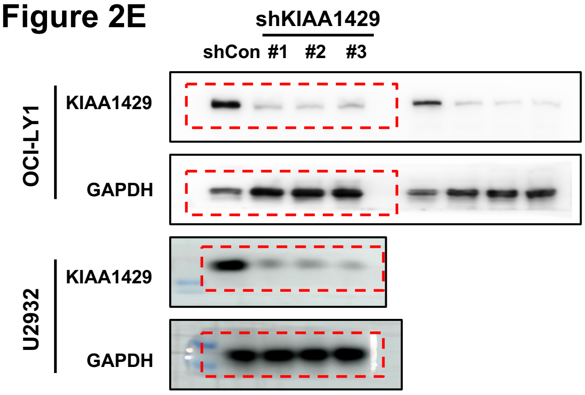

Figure 2J

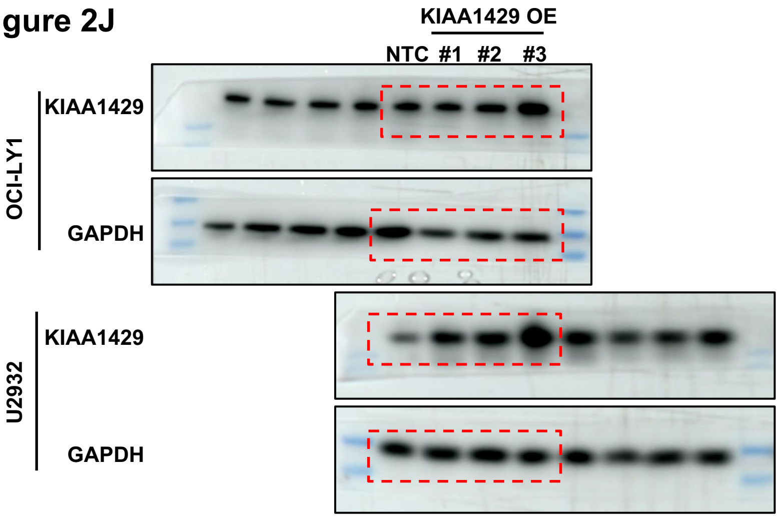

Figure 2O

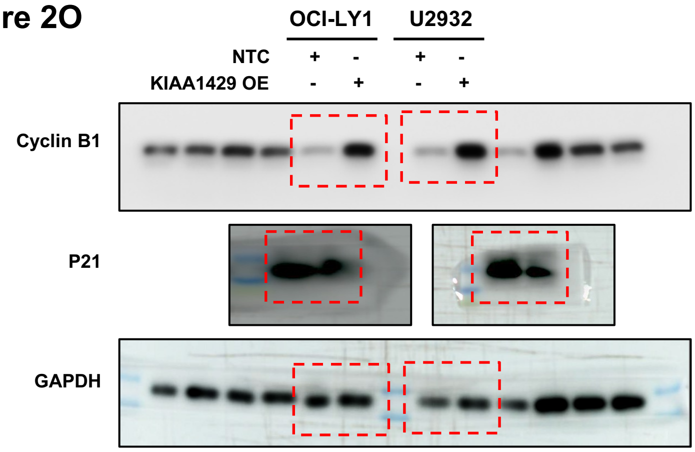

Figure 2M

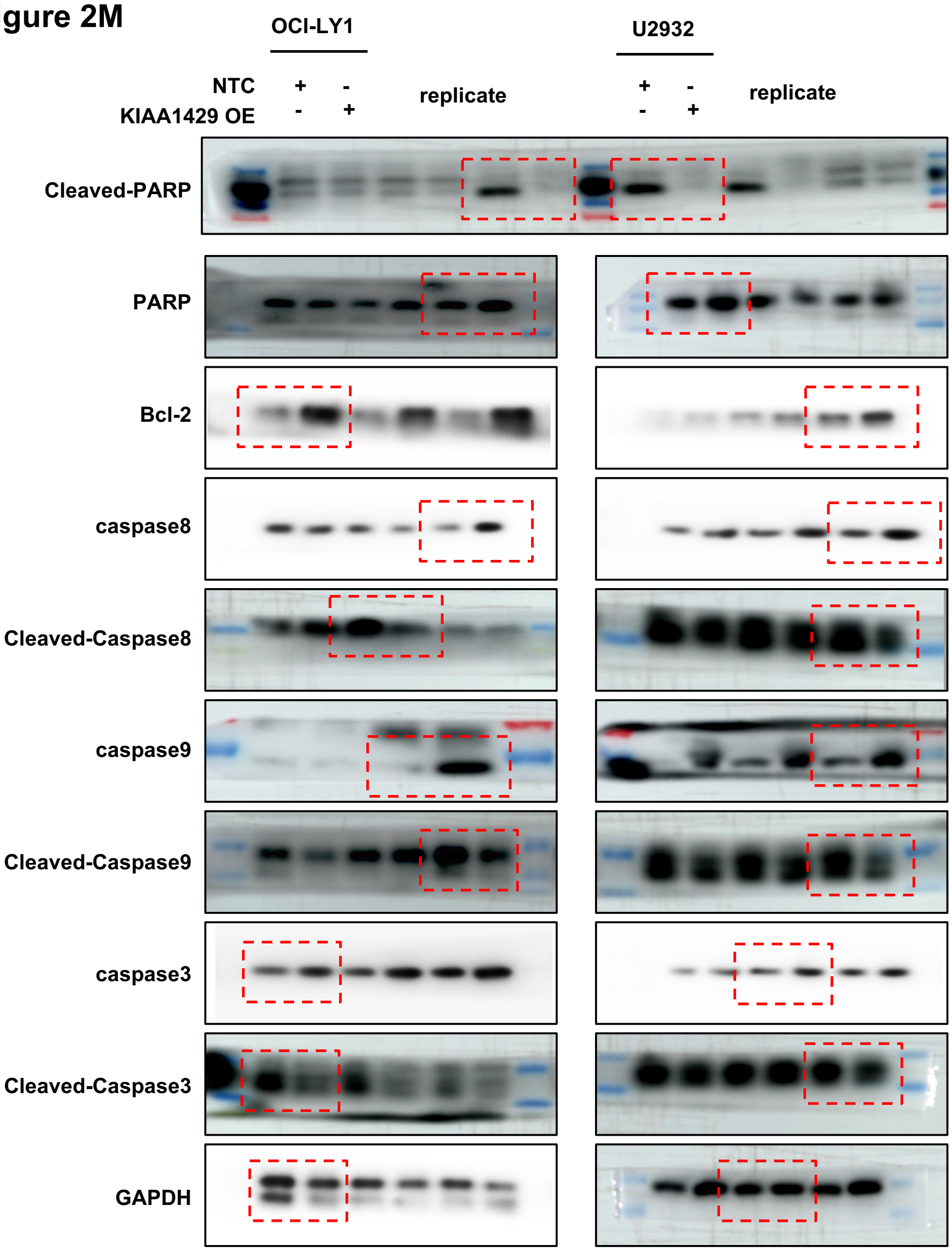
OCI-LY1
U2932
NTC + -
KIAA1429 OE - +
replicate
NTC + -
KIAA1429 OE - +
replicate
Cleaved-PARP
PARP
Bcl-2
caspase8
Cleaved-Caspase8
caspase9
Cleaved-Caspase9
caspase3
Cleaved-Caspase3
GAPDH

Figure 3B

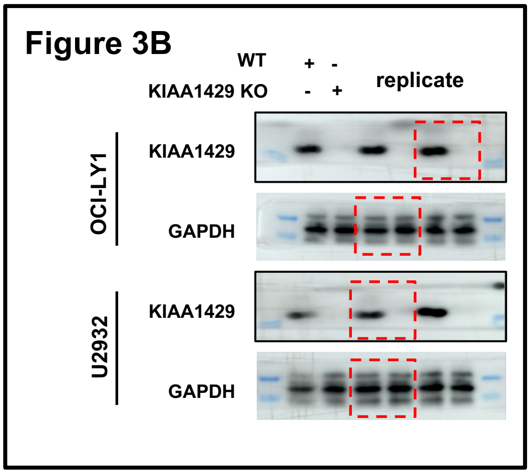

Figure 3G

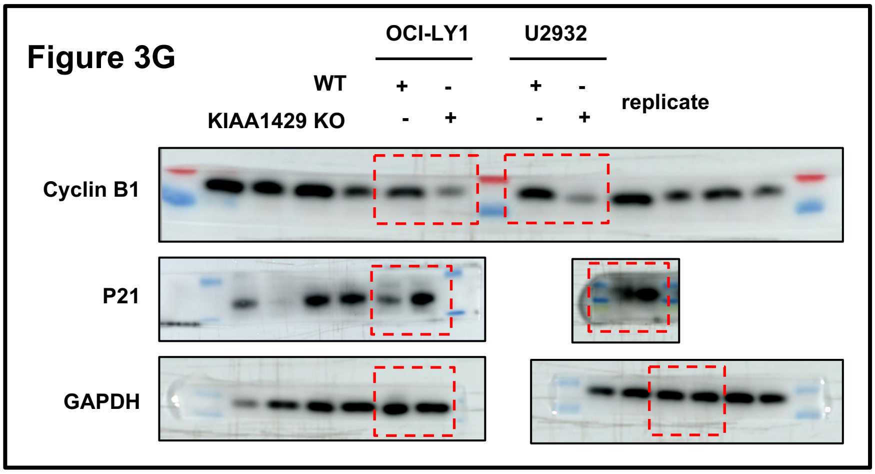

Figure 3E

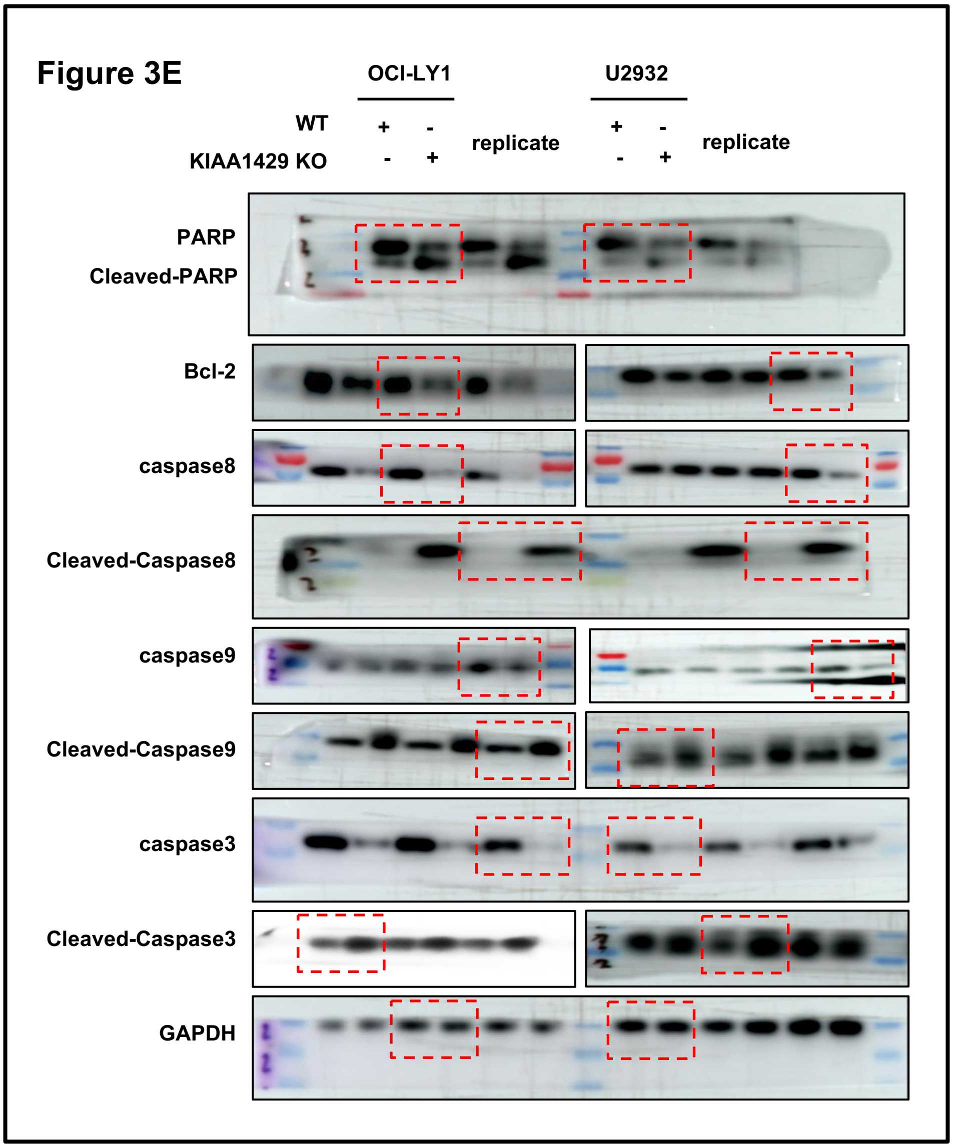

Figure 4K

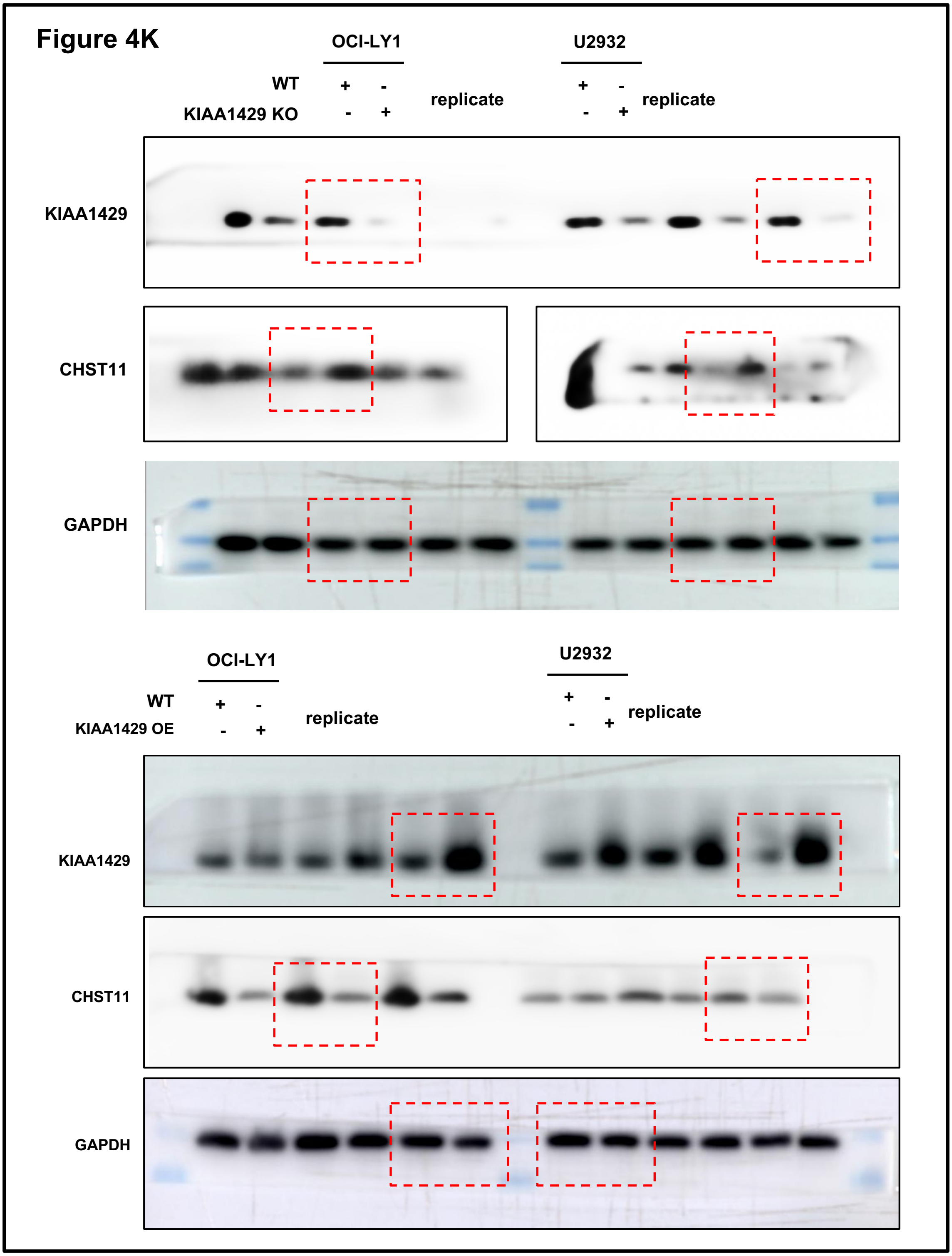

Figure 4M

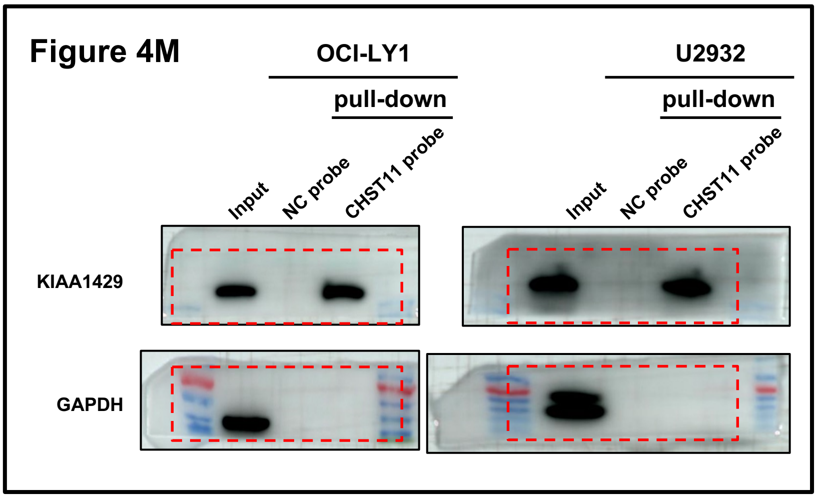

Figure 5H

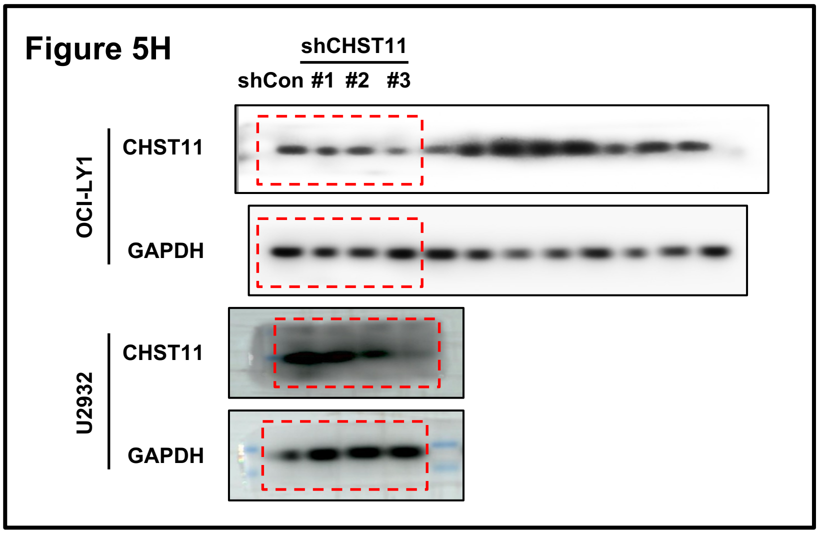

Figure 6I

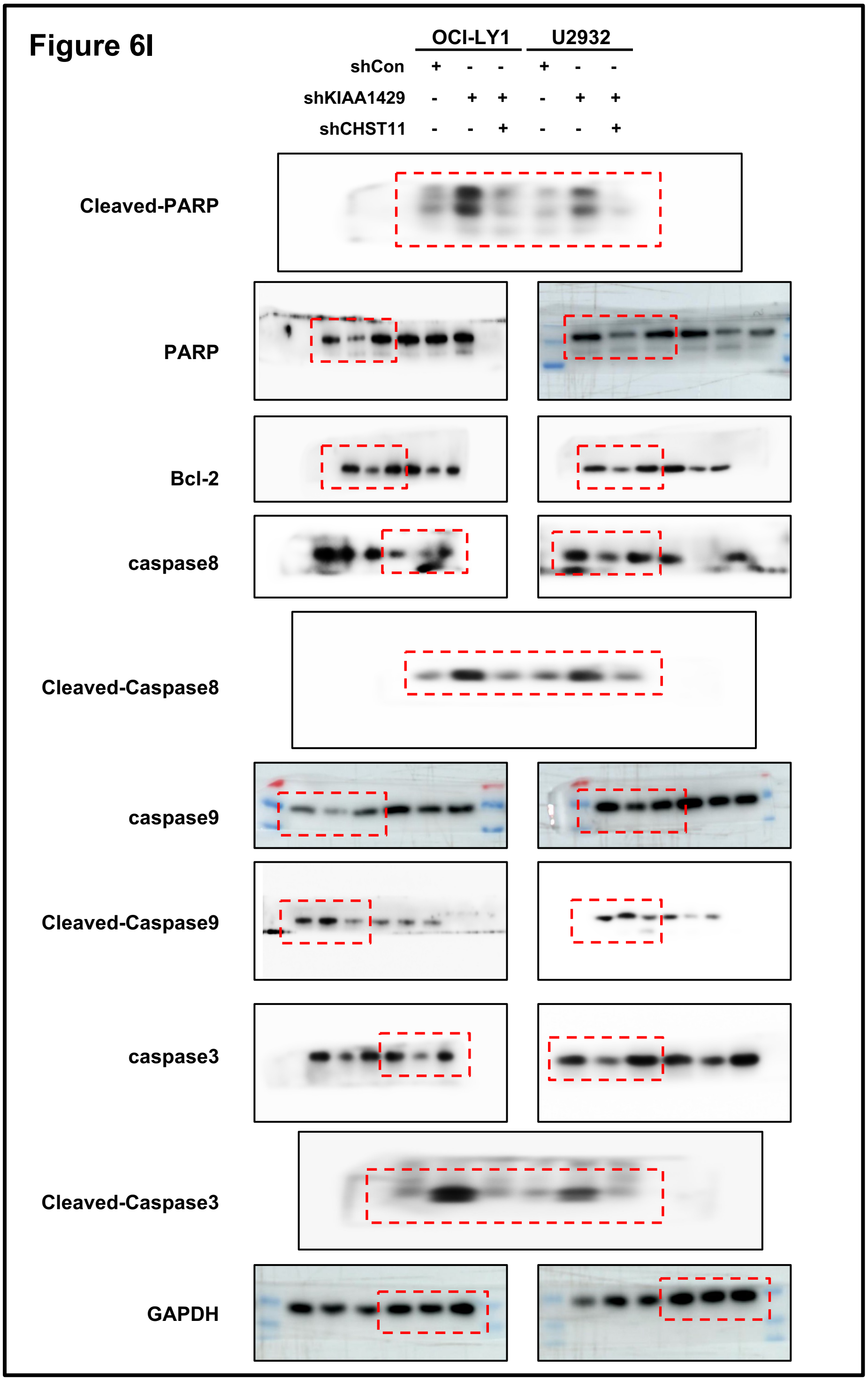

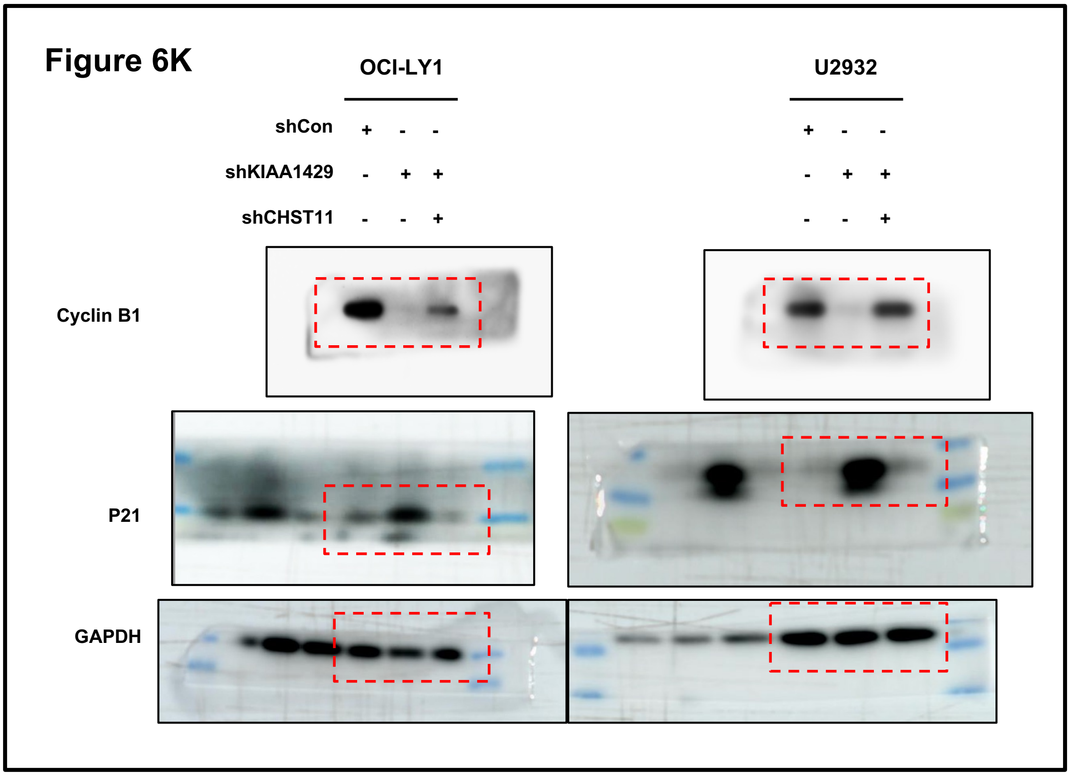
Figure 6K
OCI-LY1
U2932
shCon + - - + - -
shKIAA1429 - + + - + +
shCHST11 - - + - - +
Cyclin B1
P21
GAPDH

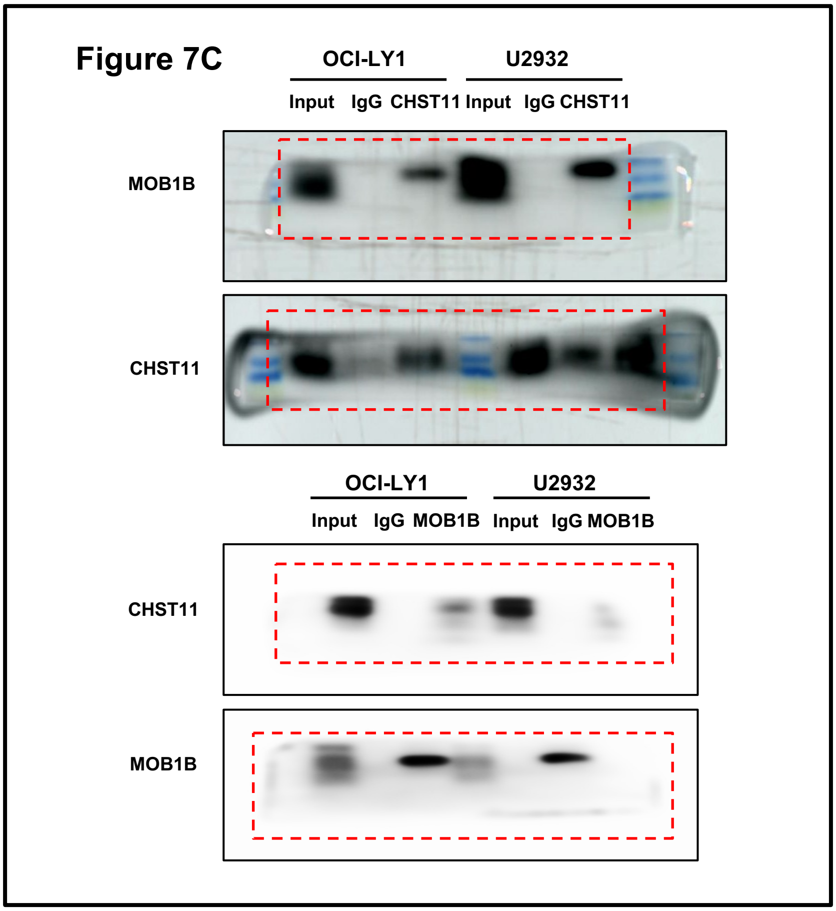
Figure 7C
OCI-LY1
U2932
Input IgG CHST11 Input IgG CHST11
MOB1B
CHST11
OCI-LY1
U2932
Input IgG MOB1B Input IgG MOB1B
CHST11
MOB1B

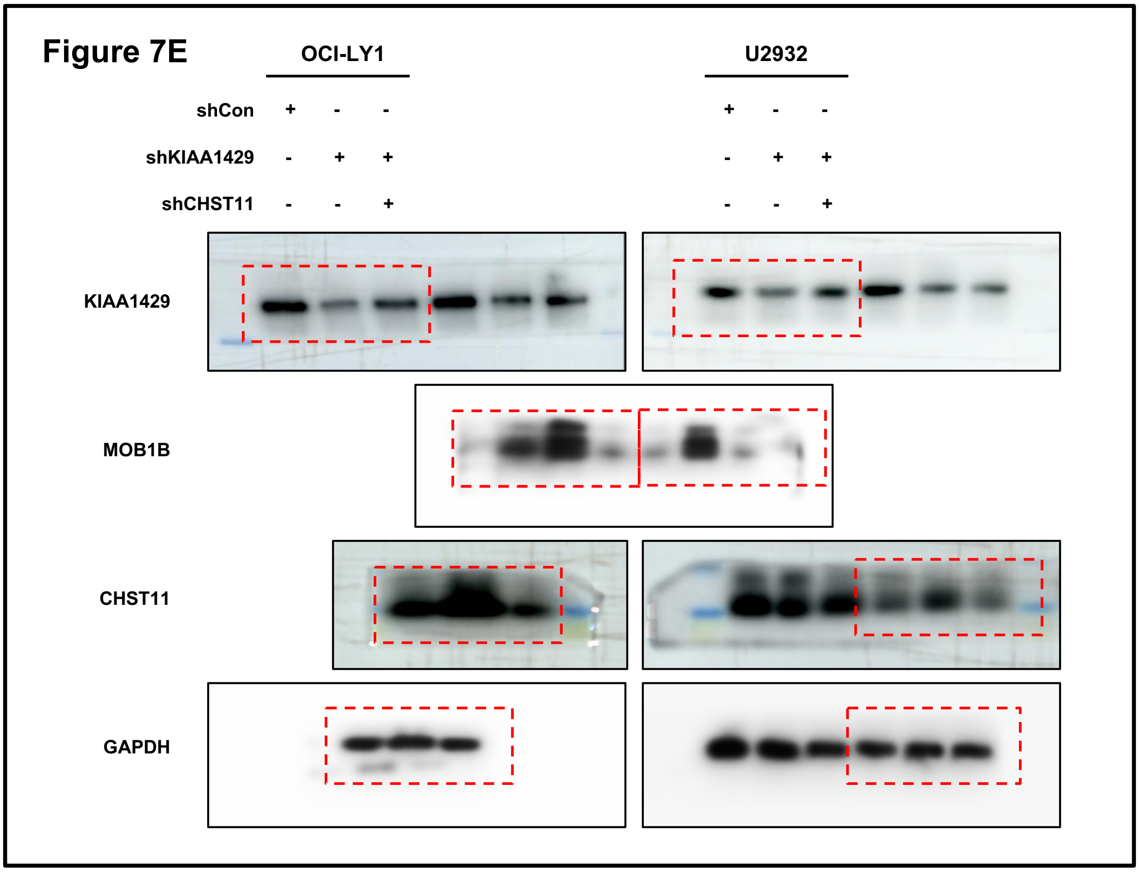
Figure 7E
OCI-LY1
U2932
shCon + - - + - -
shKIAA1429 - + + - + +
shCHST11 - - + - - +
KIAA1429
MOB1B
CHST11
GAPDH

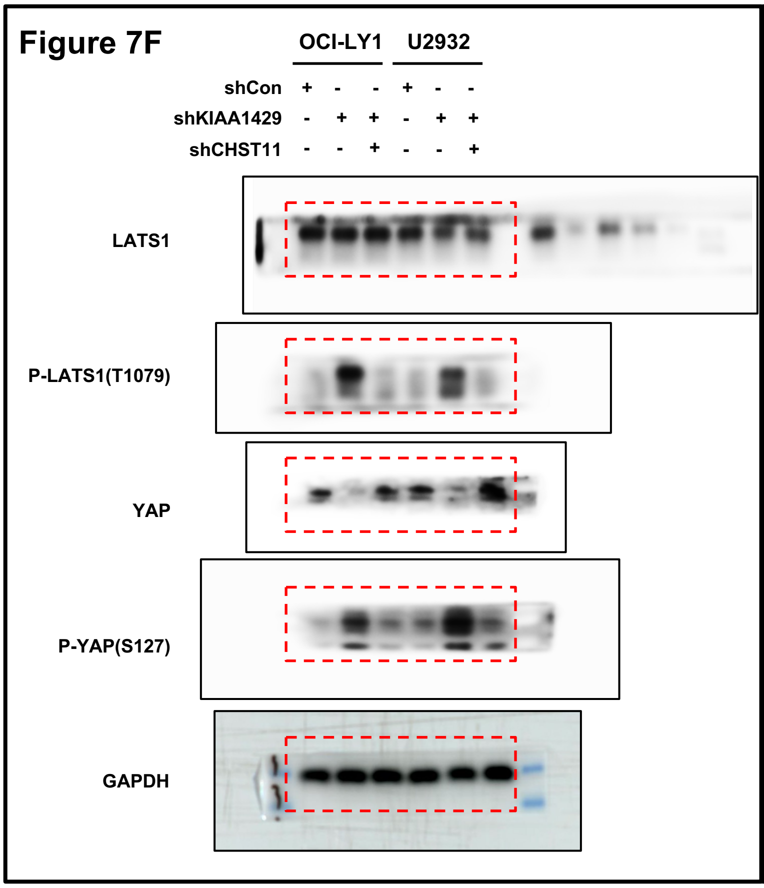
Figure 7F
OCI-LY1
U2932
shCon + - - + - -
shKIAA1429 - + + - + +
shCHST11 - - + - - +
LATS1
P-LATS1(T1079)
YAP
P-YAP(S127)
GAPDH

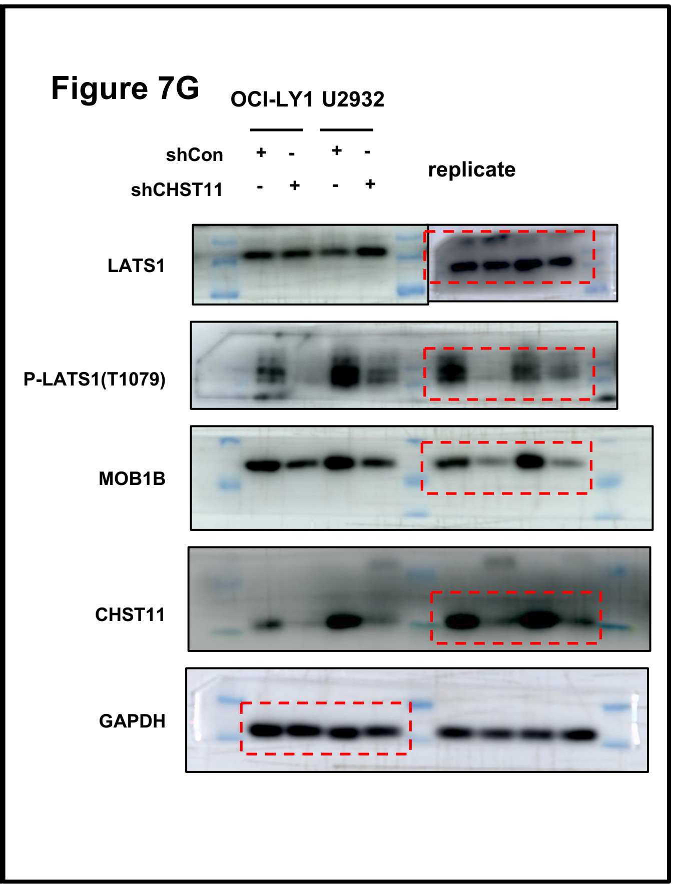
Figure 7G
OCI-LY1
U2932
shCon + - + -
shCHST11 - + - +
replicate
LATS1
P-LATS1(T1079)
MOB1B
CHST11
GAPDH

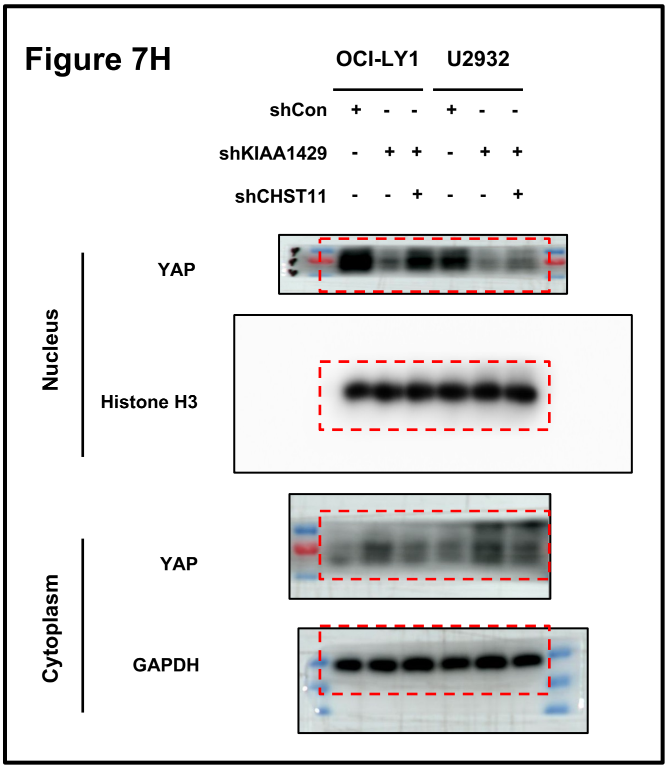
Figure 7H
OCI-LY1
U2932
shCon + - - + - -
shKIAA1429 - + + - + +
shCHST11 - - + - - +
Nucleus
YAP
Histone H3
Cytoplasm
YAP
GAPDH

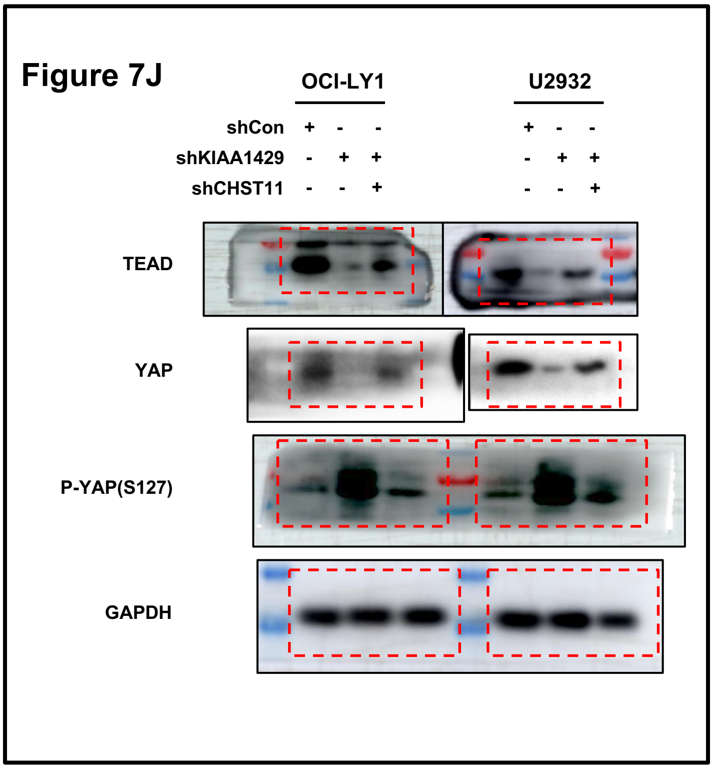
Figure 7J
OCI-LY1
U2932
shCon + - - + - -
shKIAA1429 - + + - + +
shCHST11 - - + - - +
TEAD
YAP
P-YAP(S127)
GAPDH

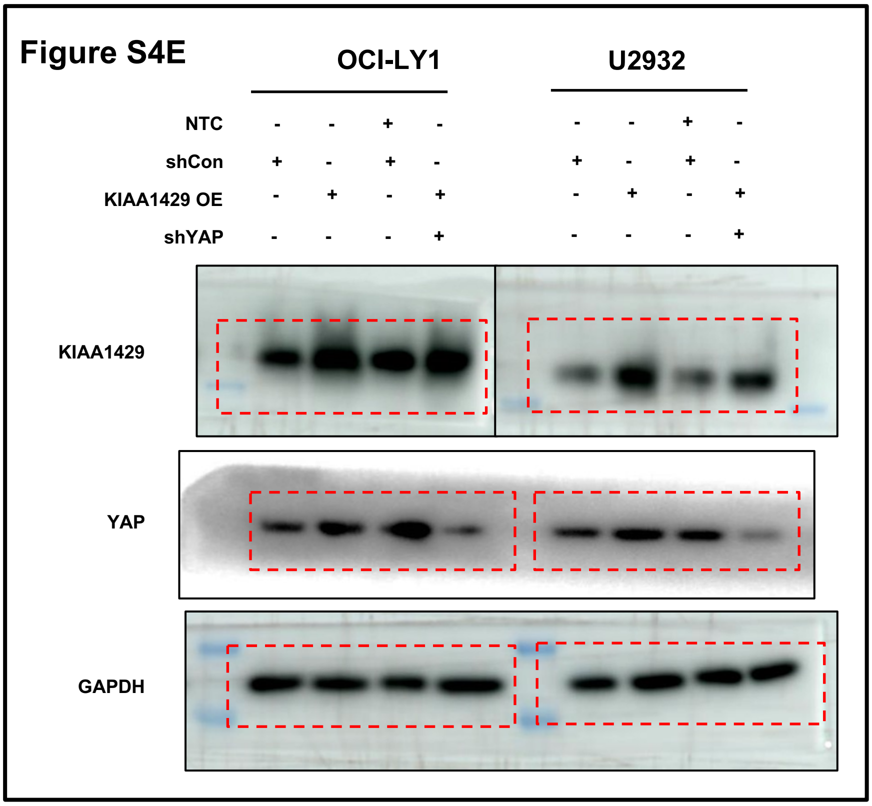
Figure S4E
OCI-LY1
U2932
NTC - - + - - - + -
shCon + - + - + - + -
KIAA1429 OE - + - + - + - +
shYAP - - - + - - - +
KIAA1429
YAP
GAPDH

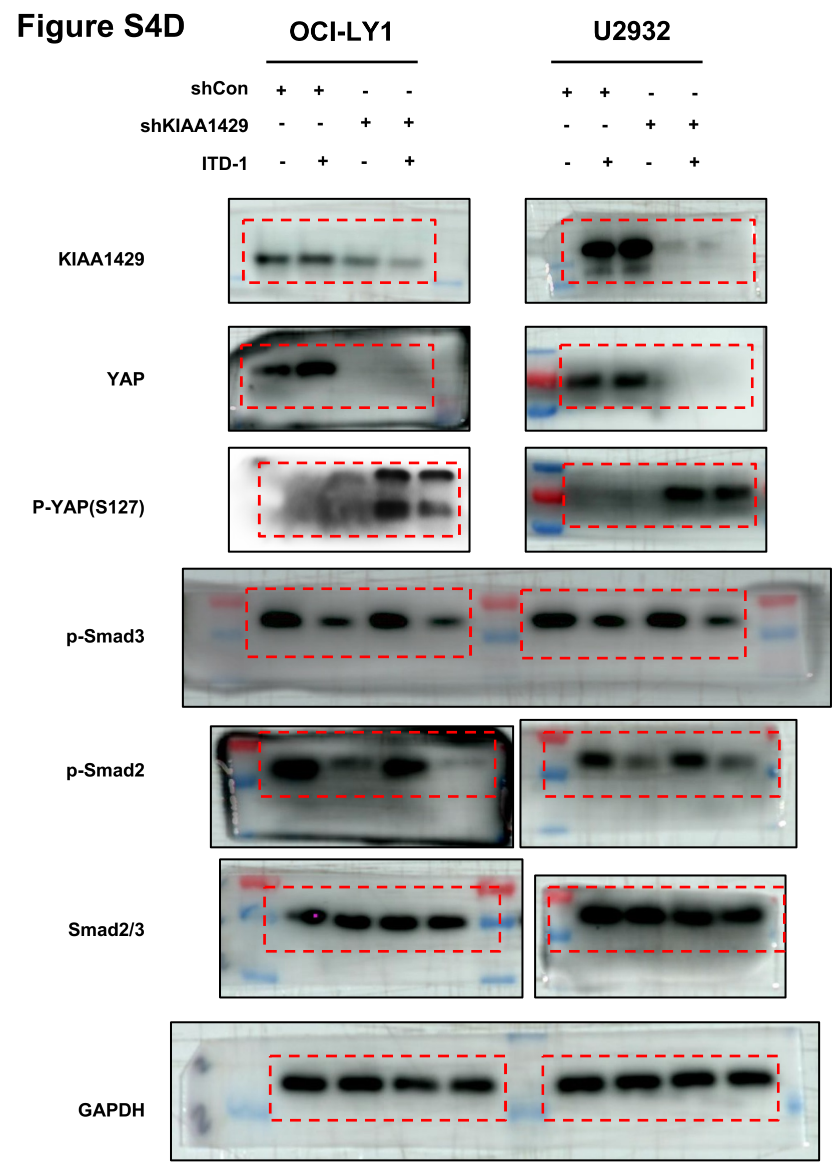
Figure S4D
OCI-LY1
U2932
shCon + + - -
shKIAA1429 - - + +
ITD-1 - + - +
shCon + + - -
shKIAA1429 - - + +
ITD-1 - + - +
KIAA1429
YAP
P-YAP(S127)
p-Smad3
p-Smad2
Smad2/3
GAPDH
